# Enhanced Catalytic Cycle of Glucose Oxidation and Reactive Species with ROS and RHS Generation Mediated by Galvanic Engineering of Dual Atomic Sites on Covalent Organic Frameworks Demonstrating Synergistic Bimetal Tumor Treatment

**DOI:** 10.1002/advs.202500515

**Published:** 2025-05-28

**Authors:** Wei‐Chung Pan, Cheng‐Hung Luo, Wen‐Ling Lin, Liu‐Chun Wang, Divinah Manoharan, Po‐Ya Chang, Hwo‐Shuenn Sheu, Chen‐Hao Yeh, Chia‐Jui Yen, Chen‐Sheng Yeh

**Affiliations:** ^1^ Department of Chemistry National Cheng Kung University Tainan 701 Taiwan; ^2^ Center of Applied Nanomedicine National Cheng Kung University Institution Tainan 704 Taiwan; ^3^ Department of Oncology National Cheng Kung University Hospital College of Medicine National Cheng Kung University Tainan 701 Taiwan; ^4^ National Synchrotron Radiation Research Center Hsinchu 30076 Taiwan; ^5^ Department of Materials Science and Engineering Feng Chia University Taichung 40724 Taiwan

**Keywords:** catalase, covalent organic frameworks, dual‐atom catalyst, galvanic replacement, glucose oxidase, haloperoxidase

## Abstract

This study introduces a novel approach to cancer treatment using covalent organic frameworks (COFs) with dual atomic metal sites, employing a galvanic reaction to integrate both gold (Au) and iridium (Ir) onto COF (COF/Au_x_/Ir_1‐x_). The integration of these metals enables a synergistic catalytic cycle that enhances glucose oxidation and the generation of reactive oxygen species (ROS) and reactive halogen species (RHS). Au catalyzes glucose oxidation, producing gluconic acid and hydrogen peroxide (H₂O₂), while Ir decomposes H₂O₂ into superoxide anion (O₂⁻) and, in the presence of chloride ions (Cl⁻), generates hypochlorous acid (HOCl). The dual metal atomic sites facilitate a feedback cycle where H₂O₂ is efficiently converted back to oxygen (O₂), amplifying ROS and RHS generation within cancer cells. By fine‐tuning the Au: Ir ratio through the galvanic reaction, optimal catalytic performance is achieved, creating a highly effective tumor treatment strategy. This work represents the first application of dual metal atomic sites on COFs for cancer therapy, demonstrating significant potential for catalysis‐based biomedical applications. The synergistic interactions between Au and Ir enhance catalytic efficiency, offering a new approach to exploiting endogenous cancer cell metabolites for targeted and efficient cancer treatment.

## Graphical abstract

A galvanic replacement is used to control dual atomic metals (Au and Ir) on a covalent organic framework (COF) nanoparticle and then coated with hyaluronic acid (HA). Under a specific bimetallic ratio (COF/Au_x_/Ir_1‐x_), the optimization can be achieved for the conversion of intracellular nutrients into reactive species (HOCl and O_2_^• ─^) and effectively inhibits the growth of liver cancer.

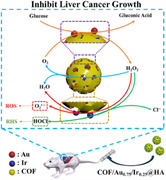

## 1 Introduction

Covalent Organic Frameworks (COFs) are crystalline, porous materials formed by linking organic molecules through strong covalent bonds.^[^ ^1^, ^2^ ^]^ These structures are synthesized using reticular chemistry, which connects carefully designed building blocks (monomers) to form extended 2D or 3D networks. COFs are highly crystalline, meaning their atomic structure is well‐ordered, which is crucial for creating uniform pore sizes and well‐defined channels. The pore sizes of COFs can be fine‐tuned during synthesis, allowing for a high degree of control over their properties. Their organic composition also enables easy functionalization, allowing the introduction of additional chemical groups or active sites to enhance or modify their performance.^[^ ^3^ ^]^ The customizable and highly ordered nature of COFs makes them valuable for a wide range of applications, including catalysis, gas storage, separation, sensing, and drug delivery.^[^ ^4^, ^5^, ^6^, ^7^, ^8^ ^]^

Dual metal atomic sites on COFs often exhibit superior catalytic activity compared to single metal sites^[^ ^9^ ^]^ due to synergistic interactions between the two metals. The proximity of these metal atoms enables cooperative catalysis, where one metal activates a substrate or intermediate, and the second metal further processes it, resulting in improved catalytic efficiency and selectivity. Additionally, the combination of two different metals allows for precise tuning of the redox properties of the catalytic sites, enhancing their performance. Common strategies for preparing dual metal atomic sites on COFs involve metalation processes,^[^ ^10^ ^]^ including the sequential or co‐addition of metal ions or ligand exchange to substitute one metal for another. Applications of COFs with dual metal atomic sites are predominantly focused on catalysis,^[^ ^11^, ^12^, ^13^, ^14^, ^15^ ^]^ including photocatalysis, where they have demonstrated exceptional performance in various reactions. The unique incorporation of two metal centers within a single framework enables cooperative catalysis, where each metal site plays a specific role in the reaction mechanism, often resulting in enhanced reaction rates and yields.

Herein, we have pioneered the creation of dual metal atomic sites on COF using a galvanic reaction, differing from the previously reported methods. Initially, IrCl_3_ was employed in a metalation process to form an Ir single atomic site on COF (COF/Ir). Due to the higher reduction potential of Au compared to Ir—where the standard reduction potential of gold (Au^3^⁺/Au) is +1.52 V,^[^ ^16^ ^]^ and that of iridium (Ir^3^⁺/Ir) is ≈+1.16 V^[^ ^16^ ^]^—the lower potential of Ir causes it to undergo oxidation, while Au ions are reduced. Through this galvanic approach, we can control the ratio of Au to Ir on COF/Au_x_/Ir_1‐x_ by adjusting the amount of AuCl₄⁻ added, thereby optimizing the composition for the best catalytic reactions.

**Figure 1:**
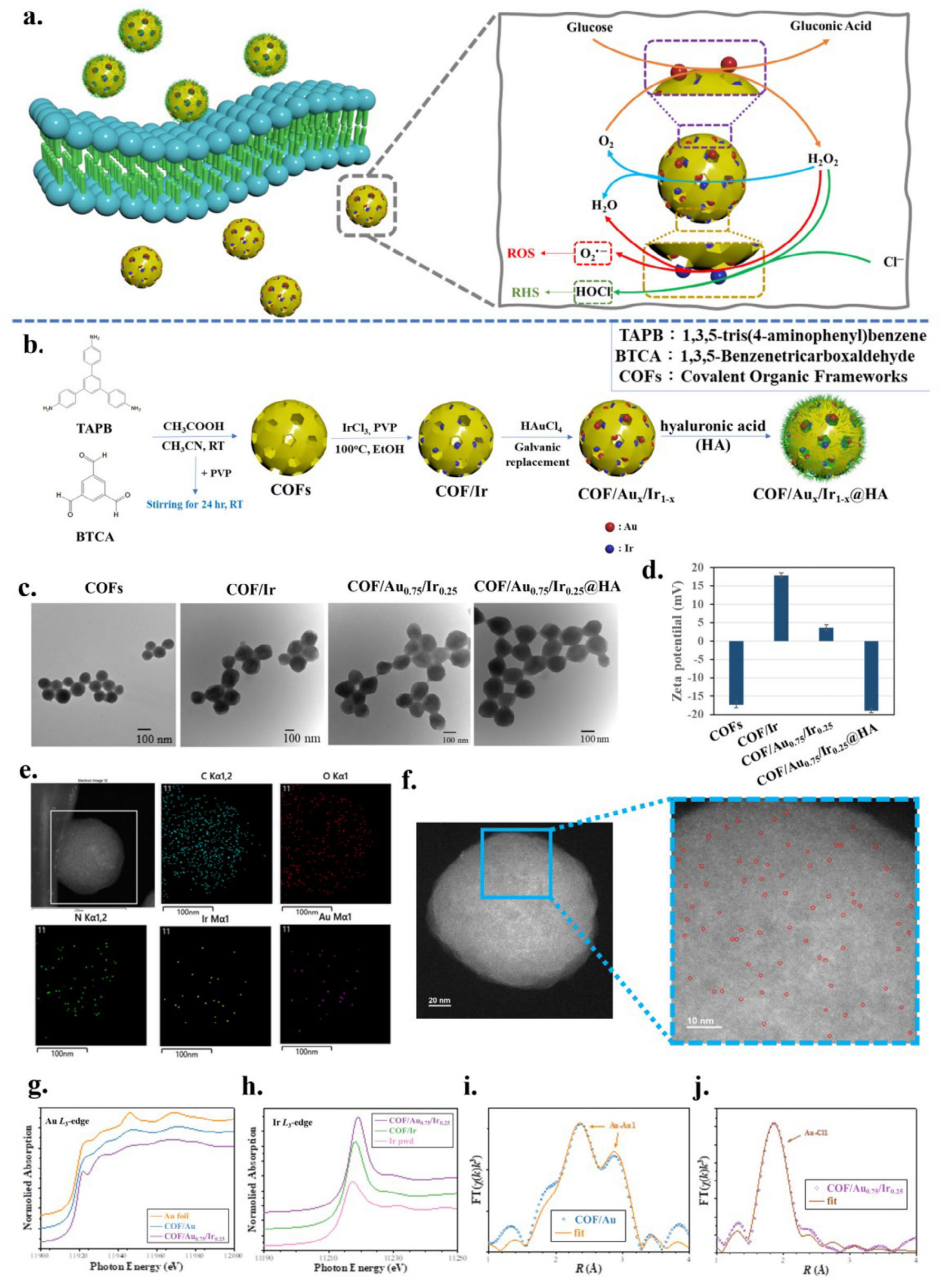
Schematic diagram and characterizations of COF/Au_0.75_/Ir_0.25_ NPs. a) Cyclic reactions of COF/Au_x_/Ir_1‐x_ including COF/Au_0.75_/Ir_0.25_ NPs by utilization of endogenous substances. b) A series of steps for synthesizing COF/Au_x_/Ir_1‐x_ including COF/Au_0.75_/Ir_0.25_ NPs. c) Transmission electron microscopy (TEM) images of COFs, COF/Ir, COF/Au_0.75_/Ir_0.25_, and COF/Au_0.75_/Ir_0.25_@HA NPs. d) Zeta potential values change follow preparation processes. e) EDX‐mapping of COF/Au_0.75_/Ir_0.25_ NPs showing elemental distribution. f) AC HAADF‐STEM image of COF/Au_0.75_/Ir_0.25_ NPs (The single atoms of Au and Ir are marked by the red circles). g) XANES spectra of Au L3‐edge for COF/Au_0.75_/Ir_0.25_ NPs. h) XANES spectra of Ir *L*_3_‐edge for COF/Au_0.75_/Ir_0.25_ NPs. i) EXAFS spectra of COF/Au NPs. j) EXAFS spectra of COF/Au_0.75_/Ir_0.25_ NPs.

Given that cancer cells contain endogenous glucose, H₂O₂, and Cl⁻, by integrating both Au and Ir atomic sites on COF, we have designed an interesting enhanced catalytic cycle showing bimetallic synergistic effect for glucose oxidation and the generation of reactive oxygen species (ROS), involving O₂⁻ and HOCl in cancer cells (**Figure** **1**a). In this cyclic catalytic reaction, in the presence of O₂, the Au site oxidizes glucose to produce gluconic acid and H_2_O_2_.^[^ ^17^, ^18^ ^]^ Meanwhile, the Ir site can catalyze the decomposition of H₂O₂ to generate O_2_⁻ (superoxide anion) in the presence of H_2_O_2_ alone.^[^ ^19^ ^]^ If both H₂O₂ and Cl⁻ are present, Ir catalyzes the reaction between H_2_O_2_ and chloride ions to produce HOCl (hypochlorous acid).^[^ ^19^ ^]^ We also found that the simultaneous presence of both Au and Ir sites can effectively convert H₂O₂ back to O₂ in subsequent reactions. Therefore, this marks the first application of nanoconstructs with dual metal atomic sites on COF in cancer treatment.

While metal–organic frameworks (MOFs) have been extensively explored for integrating dual‐metal sites to enhance catalytic performance,^[^ ^20^, ^21^, ^22^ ^]^ these platforms often face limitations in structural stability, precise control over metal‐site distribution, and compatibility with biological systems. In contrast, covalent organic frameworks (COFs) offer superior chemical robustness, tunable porosity, and π‐conjugated backbones, yet remain underutilized for engineering dual‐metallic active centers.^[^ ^23^, ^24^ ^]^ Here, we introduce a novel galvanic replacement strategy to construct well‐defined dual‐metal sites on COFs, enabling fine‐tuned spatial and compositional control that is challenging to achieve in traditional MOFs. This design allows for synergistic redox activity between the metal centers, leading to the simultaneous and cyclic generation of ROS and reactive halogen species (RHS)—a redox behavior not previously reported in either MOF or COF systems. Notably, we also report the first in vivo application of dual‐metal decorated COFs for active redox modulation within the tumor microenvironment, representing a conceptual and translational advancement over previous dual‐metal nanoplatforms.

## 2 Results

### 2.1 Characterization of COFs, COF/Ir, COF/Au, and COF/Au_0.75_/Ir_0.25_ Nanoparticles

Figure 1b shows the processes in the preparation of COFs and COF/Au_x_/Ir_1‐x_. First, the COF nanoparticles (NPs) were prepared through a typical Mannich reaction with polyvinylpyrrolidone (PVP) in acetonitrile solution to control their sizes (Figure S1, Supporting Information).^[^ ^25^ ^]^ A transmission electron microscopy (TEM) image (Figure 1c) shows the COFs NPs have a sphere‐shaped morphology. The diameters of COF NPs decreased as the volume of PVP increased (Figure S2, Supporting Information). The successful preparation of COF NPs was supported by the appearance of the characteristic protonation of imine bond peaks at 1670 cm^−1^ in Fourier transform infrared spectra (Figure S3, Supporting Information)^[^ ^26^ ^]^ and in ^13^C‐NMR spectrum (Figure S4, Supporting Information).

**Figure 2:**
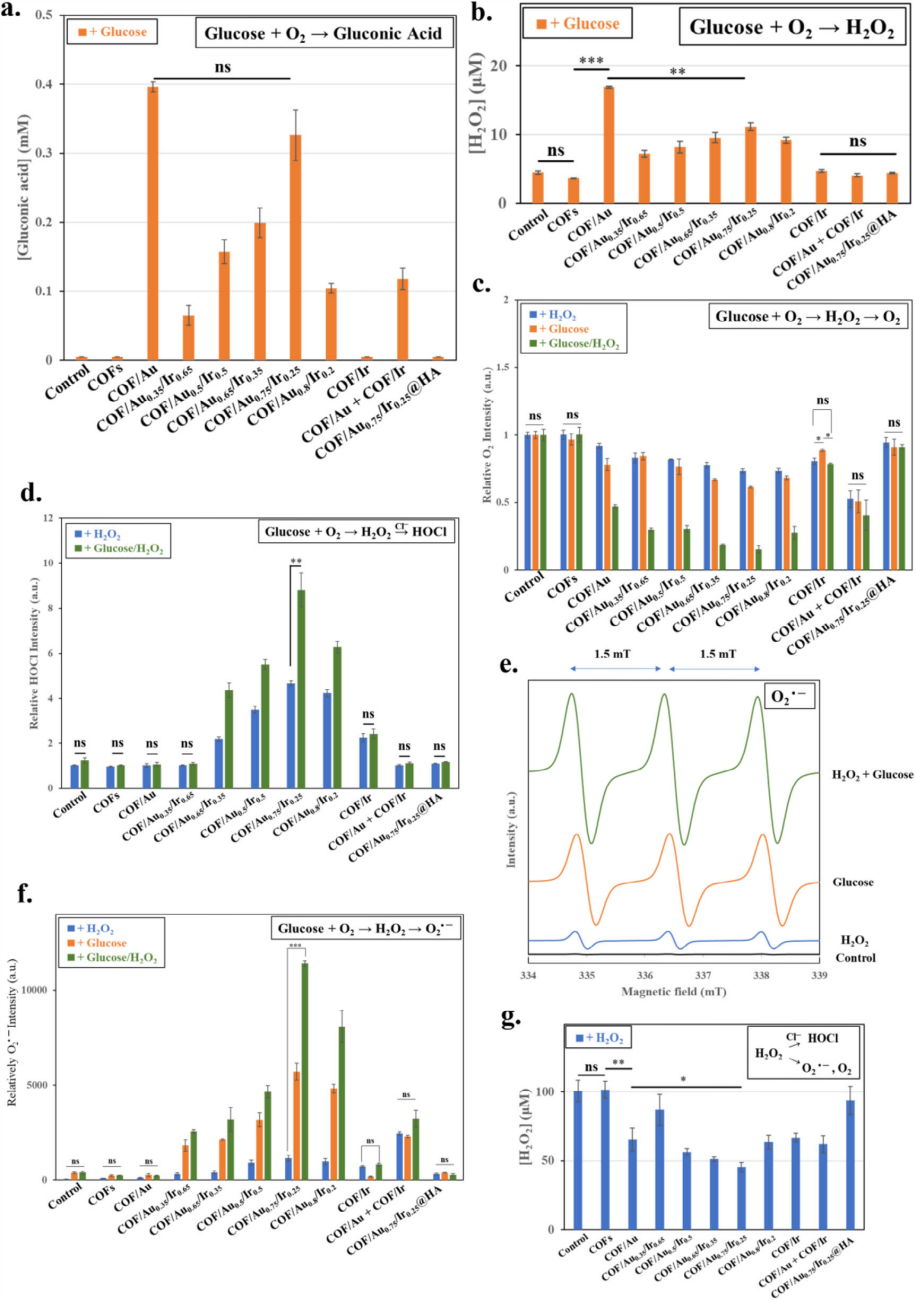
The catalytic cycle products of COFs, COF/Au, COF/Ir, COF/Au_x_/Ir_1‐x_, and COF/Au_0.75_/Ir_0.25_@HA NPs. a) Gluconic acid generation in the presence of glucose with quantified values. b) Hydrogen peroxide (H_2_O_2_) production under the presence of glucose. c) Oxygen evolution under H_2_O_2_ only, glucose alone and H_2_O_2_ + glucose groups. d) Hypochlorous acid (HOCl) formation after the addition of H_2_O_2_ alone or glucose + H_2_O_2_. e) Superoxide anion (O_2_^·−^) creation of the COF/Au_0.75_/Ir_0.25_ NPs with the 1:1:1 intensity of ESR signal under different conditions. f) The quantified ESR spectra showing liberation of O_2_^·−^ under different situations for a series of NPs. g) Consumption of H_2_O_2_ under endogenous 100 °m H_2_O_2_.

Before producing dual Au and Ir atoms on COFs (COF/Au_x_/Ir_1‐x_), we first synthesized single‐atom COF/Ir and COF/Au separately to understand their catalytic behaviors. The size of 75 nm was chosen to dope Ir atoms on COFs, given the COF/Ir NPs. The various amounts of Ir atoms on COFs were synthesized through an alcoholic reduction of different volumes of IrCl_3(aq)_ in the presence of PVP.^[^ ^27^ ^]^ After treatments, we observed the sizes of COFs and COF/Ir NPs increased to 150 nm without forming apparent Ir NPs in TEM images (Figure 1c; Figures S5 and S6, Supporting Information). COFs maintain a size of 150 nm, unaffected by the amount of Ir salt added. The zeta potential switches from −17.3 to +17.9 mV (Figure 1d). The high‐resolution TEM and aberration corrected high‐angle annular dark‐field scanning transmission electron microscopy (AC‐HAADF‐STEM) were further used to explore the feature of COF/Ir. Importantly, the AC‐HAADF‐STEM with the atomic resolution confirms atomically Ir atoms (marked by red circles) bearing onto the COFs under the low volumes of IrCl_3(aq)_ (Figure S7, Supporting Information). When the amount of IrCl_3(aq)_ added was 20 µL, we found that it had the highest Ir single‐atom distribution on the COF. On the other hand, when the addition amount of IrCl_3(aq)_ exceeded 50 µL, the COF/Ir NPs exhibited Ir (111) lattice planes, indicating the formation of larger Ir particles (Figure S7, Supporting Information). Figure 2c shows the TEM of COF/Ir using 20 µL of IrCl_3(aq)_. Next, we evaluate the catalytic behavior of COF/Ir by the measurement of the hydroxyl radical (·OH) generation. The detection of ·OH follows the observation of fluorescence emission (*λ*_em_ = 425 nm) using terephthalic acid (TPA) as a probe under simulated endogenous hydrogen peroxide (H_2_O_2_) conditions (100 °m) (Figure S8, Supporting Information). All groups show no ·OH generated. However, HOCl generation of COF/Ir increases as the concentrations ([Cl^−^]: 0, 40, and 140 mm) of chloride ions rise in the presence of H_2_O_2_ (100 °m) (Figure S9, Supporting Information) with COF/Ir (20 µL) behaving highest HOCl yield. 40 mm of Cl^−^ follows the endogenous concentration, while 140 mm of Cl^−^ is the concentration in PBS. Aminophenyl fluorescein solution (APF) was used to specifically detect HOCl. The electron spin resonance (ESR) demonstrates COF/Ir can further decompose H_2_O_2_ into O_2_^·−^. The trapping agents of 1‐hydroxy‐3‐methoxycarbonyl‐2,2,5,5‐tetramethylpyrrolidine (CMH) were used to sense O_2_^·−^. The Ir concentration of COF/Ir was fixed at 10 ppm to obtain approximately close ESR signal intensity from different groups of COF/Ir. We found that O_2_^·−^ is generated only when H_2_O_2_ is added in all the COF/Ir and Ir groups, indicating that O_2_^·−^ is derived from H_2_O_2_ (Figure S10, Supporting Information).

Literature has demonstrated Au NPs showing glucose oxidase catalytic behavior.^[^ ^17^, ^18^ ^]^ Here, we would like to examine Au atom‐doped COF (COF/Au NPs) performing enzymatic oxidation reactions similar to glucose oxidase. After coordinating the Au ions by adding aqueous HAuCl₄ to the COFs, Au⁰ is deposited onto the COF through reduction with NaBH₄. In this experiment. We simultaneously synthesized Au atoms and Au NPs, and then tried to understand the impact of Au atoms and Au NPs on the efficiency of enzymatic oxidation reactions. Although Au NPs can be observed in the TEM images (Figures S11 and S12, Supporting Information), the AC‐HAADF‐STEM images show that the COF/Au NPs still contain single‐atom Au (Figure S13, Supporting Information). When the amount of HAuCl_4(aq)_ added was 1 µL, we found that it had the highest Au single‐atom distribution on the COF. On the other hand, when the amount of aqueous HAuCl₄ added reaches 5 µL, the COF/Au exhibits Au (111) and Au (200) lattice planes, indicating the formation of Au NPs. In the XRD pattern, as the amount of Au salt increases, the distinct Au characteristic peaks become evident when the amount of HAuCl_4(aq)_ is up to 5 µL. (Figure S14, Supporting Information). We used a known method by mixing hydroxylamine and Fe (III) to detect gluconic acid (Figures S15 and S16, Supporting Information).^[^ ^17^, ^28^ ^]^ The results show that the sample with the highest amount of single‐atom Au has the highest gluconic acid yield. It was also found that the more Au NPs formed on the COFs, the lower the efficiency of glucose to gluconic acid conversion. This result demonstrates that Au atoms exhibit excellent glucose oxidase‐like enzymatic oxidation behavior (Figure S17, Supporting Information).

After understanding the individual catalytic abilities of COF/Ir and COF/Au, Au and Ir dual atoms were then deposited on the COFs to form COF/Au_x_/Ir_1‐x_. Due to the higher reduction potential of Au compared to Ir, COF/Ir was used as a template to form dual Au and Ir atoms via the galvanic replacement process. We selected COF/Ir (IrCl_3_: 20 µL) NPs, which exhibited the highest atomic distribution and the greatest HOCl production, and obtained bimetallic COF/Au_x_/Ir_1‐x_ NPs. With the addition of less than 100 µL of HAuCl_4(aq)_, no NP formation on COFs was observed in TEM images (Figure 1c; Figure S18, Supporting Information). Figure 1c presents a COF/Au_0.75_/Ir_0.25_ image showing the greatest catalytic behavior, which will be shown later. The AC‐HAADF‐STEM images show that when the volume of Au salt added reaches 100 µL, not only do Au atoms form, but Au NPs also begin to appear. When the volume of Au salt reaches 200 µL, only Au NPs are present, and no Ir signal is detected (Figure S19, Supporting Information). EDX‐mapping measurements display that carbon, nitrogen, oxygen, Ir, and Au elements are distributed throughout the COF for COF/Au_0.75_/Ir_0.25_ (Figure 1e). The AC‐HAADF‐STEM images display the single atoms of Au and Ir marked by the red circles on COF (Figure 1f). The XPS measurements reveal both Au and Ir elements on COF/Au_0.75_/Ir_0.25_ (Figure S20, Supporting Information).

Using X‐ray absorption spectroscopy, we can investigate the chemical environment surrounding metal atoms, which helps us understand the coordination of metal ions and the resulting chemical structure. The Au *L*_3_‐edge XANES spectra (Figure 1g) reveal different results for the COF/Au and COF/Au_0.75_/Ir_0.25_ samples. The white line of the COF/Au_0.75_/Ir_0.25_ sample at 11 922 eV is prominent compared to that of the Au foil and COF/Au sample. This white line corresponds to the electronic transition from the 2p3/2 core‐level state to an unoccupied 5d state. In contrast, the white line is not observed in Au foil, as the 5d states in Au foil are fully occupied by d‐electrons.^[^ ^29^ ^]^ Based on the results from Alberto Villa et al.,^[^ ^30^ ^]^ we can estimate the valence of Au in COF/Au_0.75_/Ir_0.25_ to be ≈+1, as indicated by the white line intensity.

COF/Ir and COF/Au_0.75_/Ir_0.25_ exhibit a white line in the Ir *L*_3_‐edge spectra (Figure 1h) compared to Ir powder, with the COF/Au_0.75_/Ir_0.25_ sample showing a higher intensity and a slight shift to higher photon energy. This shift indicates an increasing in the oxidation state of the Ir ions. According to Alessandro et al. and Hong et al.,^[^ ^31^, ^32^ ^]^ the white line of IrCl_3_ is approximately at 11 217.3 eV and for IrO_4_, it is ≈11 218.8 eV. By analyzing the position of the white line, the oxidation state of COF/Ir, positioned at 11 218.3 eV, appears to be larger than +3. Conversely, the white line at 11 219.1 eV in COF/Au_0.75_/Ir_0.25_ indicates an oxidation state greater than +4.

The frequency of oscillations observed for Au foil and the COF/Au sample are similar, indicating that the COF/Au sample retains the Au face‐centered cubic (fcc) structure (Figure S21, Supporting Information). Fitting the Fourier Transforms (FT) at the Au *L*₃‐edge provides more detailed information about the surrounding atoms and coordination numbers. According to the results shown in Figure 1i and Table S1 (Supporting Information), the Au atoms in the COF/Au sample are primarily surrounded by other Au atoms, with a coordination number of 9.24. The distance between two Au atoms is ≈2.79 Å, slightly shorter than the 2.86 Å observed in Au foil, suggesting the formation of short‐range Au–Au nanoparticles.^[^ ^33^ ^]^

In contrast, the FT curve of COF/Au_0.75_/Ir_0.25_ is best fitted by the Au‐Cl path, indicating that Au atoms form clusters with Cl atoms, with a coordination number of 2.05, rather than with other Au atoms (Figure 1j, Table S2, Supporting Information). Additionally, the absence of an Au‐Ir path confirms that Au and Ir atoms are isolated in the dual‐atom system. From the coordination number, we can estimate the valence of the Au ion to be +1. In COF/Au_0.75_/Ir_0.25_, Ir atoms tend to reach a high oxidation state, which enables Au atoms to easily obtain electrons from Ir atoms. This result shows that the addition of HAuCl₄ (Au^3^⁺) initiates a galvanic reaction, creating an Au¹^+^ and Ir⁴⁺ oxidation state on the COFs. It is worth noting that when an excess of HAuCl₄ (200 µL) is added, the Ir signal cannot be detected on the COFs (Figures S18 and S19, Supporting Information).

### 2.2 Catalytic Cyclic Reactions of COF/Au_0.75_/Ir_0.25_ NPs

We investigate the catalytic behavior of the Au: Ir dual‐atom system with varying ratios in COF/Au_x_/Ir_1‐x_. Our initial focus is on their glucose oxidase‐like catalytic reaction. It is well‐established that Au NPs can catalyze the conversion of glucose into gluconic acid and H₂O₂.^[^ ^34^ ^]^ Au first adsorbs glucose and oxygen onto its surface, then catalyzes the oxidation of the aldehyde group (─CHO) to a carboxyl group (─COOH), resulting in gluconic acid. At the same time, O_2_ is reduced to H_2_O_2_. To measure the amount of gluconic acid produced, we first reacted hydroxylamine with gluconic acid to form a hydroxamate‐Fe^3^⁺ complex with Fe (III), and detected the maximum absorbance at 505 nm. All the groups have a fixed concentration of [Au]_10 ppm_. An increase in absorbance at 505 nm indicated higher gluconic acid production, as evidenced by varying degrees of color change in solutions containing COF/Au and COF/Au_x_/Ir_1‐x_ NPs, with no change observed in COFs or COF/Ir. The quantitative analysis of gluconic acid response intensity is shown in **Figure** **2**a. COF/Au₀.₇₅/Ir₀.₂₅ NPs exhibited the highest gluconic acid production, comparable to COF/Au, and significantly higher than the other groups. In contrast, COFs and COF/Ir NPs did not catalyze glucose and therefore did not produce gluconic acid. Additionally, gluconic acid production from COF/Au₀.₇₅/Ir₀.₂₅ NPs surpassed that of the physically mixed COF/Au and COF/Ir group, suggesting a synergistic effect within the bimetallic system. Since COF/Au initially showed a high gluconic acid yield and COF/Ir did not catalyze glucose oxidation, it was originally expected that physically mixing COF/Au with COF/Ir would result in a similar yield of gluconic acid as COF/Au. However, contrary to expectations, this physical mixing resulted in decreased gluconic acid production, indicating an antagonistic effect.

The generation of H₂O₂ was quantified by the increased fluorescence emission (*λ*_em_ = 515 nm) using a hydrogen peroxide assay kit (Figure 2b). Upon the addition of glucose, COF/Au and COF/Au₀.₇₅/Ir₀.₂₅ exhibited the highest H₂O₂ production, while COFs and COF/Ir did not show this capability. Notably, compared to the results in Figure 2a, the amount of H₂O₂ generated did not directly correlate with the amount of glucose added and COF/Au₀.₇₅/Ir₀.₂₅ reveals less amount of H_2_O_2_ than that of COF/Au, suggesting the presence of additional pathways for H₂O₂ consumption.

Accordingly, we further investigated the catalase‐like enzymatic reaction, which involves converting H₂O₂ into O₂. The O₂ fluorescent sensing agent [Ru(dpp)₃]Cl₂ was used to observe the decrease in fluorescence intensity at 618 nm in the presence of O₂. Since [Ru(dpp)₃]Cl₂ exhibits a decrease in fluorescence signal upon encountering O₂, our experiments aim to observe whether these COFs containing metal will exhibit catalase‐like activity. Therefore, before conducting the experiments, we first purge the solution system with N₂ to minimize the interference of O₂ present in the solution. After purging, under conditions with minimal O₂, we tested and found that both COF/Au and COF/Au_x_/Ir_1‐x_ could convert H₂O₂ into O₂, with COF/Au₀.₇₅/Ir₀.₂₅ showing the highest efficiency. However, COF/Ir could only decompose H₂O₂ into O₂, and even with the addition of glucose, the amount of O₂ produced did not increase. COFs alone do not have catalase‐like behavior showing no O_2_ production (Figure 2c). All the groups have fixed concentrations of [Au]_10 ppm_ and [Ir]_10 ppm_. Clearly, COF/Au₀.₇₅/Ir₀.₂₅ has the highest capability to participate in a cyclic reaction with glucose to form gluconic acid and H₂O₂, and then convert H₂O₂ back into O₂. Although glucose oxidation consumes oxygen, the synergistic effect of the bimetallic system accelerates the conversion of H_2_O_2_ to O_2_, allowing for the detection of O_2_ production.

Ir exhibits haloperoxidase (HPO) activity. It first binds with H₂O₂ and undergoes dehydration, forming a bond between oxygen and Cl⁻. This is followed by hydrolysis and the release of HOCl. Additionally, Ir can adsorb H₂O₂ molecules and decompose them into two OH radicals. These OH radicals then react separately with protons and H₂O₂ to produce H₂O and O₂^•^⁻.^[^ ^19^ ^]^ We used aminophenyl fluorescein (APF) as an indicator for HOCl, which increases fluorescence intensity (*λ*_em_ = 515 nm) in the presence of HOCl. All the groups have fixed concentrations of [Au]_10 ppm_ and [Ir]_10 ppm_. COF/Ir and COF/Au_x_/Ir_1‐x_ serve as chloride‐driven catalysts. In the PBS solution used for measurements, Cl⁻ is sourced from the PBS and reacts with H₂O₂ to produce HOCl. When H₂O₂ and glucose are present together, the generation of HOCl is significantly enhanced in the COF/Au₀.₇₅/Ir₀.₂₅ group due to the effective cooperation between Au and Ir (Figure 2d). Besides, all groups show no ·OH generated through TPA dye (Figure S22, Supporting Information).

O_₂_^•^⁻ was detected using CMH through ESR spectral analysis, with a characteristic peak ratio of 1:1:1 indicating the presence of O₂^•^⁻. The highest O₂^•^⁻ production was observed in COF/Au₀.₇₅/Ir₀.₂₅, ranked from lowest to highest as follows: control group, H₂O₂, glucose, and H₂O₂ + glucose (Figure 2e). All the groups have fixed concentrations of [Au]_10 ppm_ and [Ir]_10 ppm_. The quantification of ESR signals for different COFs is shown in Figure 2f. No O₂^•^⁻ was detected in COFs and COF/Au, in contrast to COF/Ir and COF/Au_x_/Ir_1‐x_. For COF/Ir, Ir reacts with only a limited amount of H₂O₂ to produce O₂^•^⁻, but does not react with glucose. In contrast, COF/Au₀.₇₅/Ir₀.₂₅ with H₂O₂ + glucose showed approximately twofold higher O₂^•^⁻ generation compared to the glucose‐only group, and tenfold greater than the H₂O₂‐only group.

We also present the quantitative measurement of H₂O₂ consumption (Figure 2g). Using a hydrogen peroxide assay kit, we calculated the remaining H₂O₂ content after NPs interacted with 100 °m of H₂O₂. All groups, except for COFs alone, demonstrated the ability to deplete H₂O₂. The COF/Au₀.₇₅/Ir₀.₂₅ group exhibited the highest degree of H₂O₂ consumption, leaving only 45.5 °m of H₂O₂ remaining after 30 min.

Collectively, these results reveal that COF/Au₀.₇₅/Ir₀.₂₅ exhibits the highest catalytic activity for various enzymatic function mimics due to the bimetallic synergistic effect in cyclic reactions.

### 2.3 In Vitro Cytotoxicity in COF@HA, COF/Au@HA, COF/Ir@HA, and COF/Au_0.75_/Ir_0.25_@HA NPs

From the previous catalytic behavior tests, we already know that COF/Au₀.₇₅/Ir₀.₂₅ exhibits the best catalytic performance, so we proceeded with COF/Au₀.₇₅/Ir₀.₂₅ as the sample for further in vitro and in vivo studies. To prevent COF/Au₀.₇₅/Ir₀.₂₅ from initiating a series of catalytic reactions while flowing through the vessels before reaching the tumor during in vivo experiments, we modified the surface of COF/Au₀.₇₅/Ir₀.₂₅ with hyaluronic acid (HA) (Figure 1c). Another purpose of modifying with HA is that HA can specifically bind to the CD44 receptor.^[^ ^35^ ^]^ The overexpression of CD44 in cancer cells would enable targeted therapy.^[^ ^36^ ^]^ Figure S23 (Supporting Information) shows the FTIR spectra, where the characteristic C─O bond peak at 1041 cm⁻¹ confirms the successful modification of HA on the surface of the NPs. After HA modification, the zeta potential of the NPs shifts from positive to negative (Figure 1d). Additionally, the hydrodynamic diameter of COF/Au₀.₇₅/Ir₀.₂₅@HA increases (Figure S24, Supporting Information). The HA‐modified COF/Au₀.₇₅/Ir₀.₂₅ remains stable without aggregation after 7 days of incubation different physiological conditions (H₂O, PBS: pH 5, PBS: pH 7, and 10% fetal bovine serum (FBS) medium in Figure S25, Supporting Information). More importantly, COF/Au₀.₇₅/Ir₀.₂₅@HA does not undergo a series of cyclic reactions, ensuring that no reactions occur during vessel flow before reaching the tumor site in the in vivo animal experiments. After HA homes in the cancer cells, it can be stripped off, either through enzymatic degradation (e.g., by hyaluronidase) or by diffusion across the cell membrane, thereby exposing the NPs for subsequent processes.^[^ ^37^ ^]^ In the following experiments, including MTT assay, flow cytometry, cell imaging, and animal studies, the NPs used are all coated with HA.

**Figure 3:**
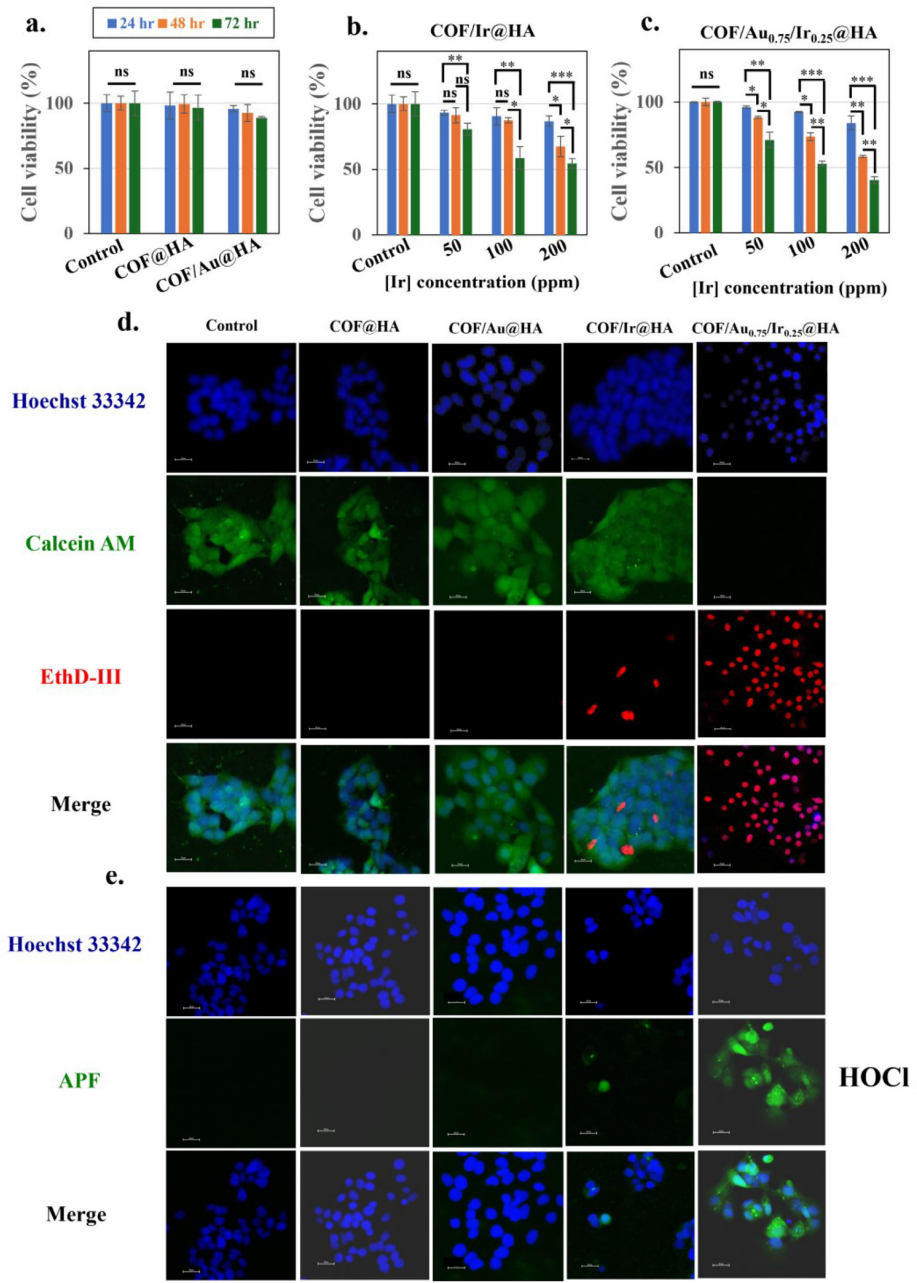
The in vitro examination of HepG2 cells incubated with COF@HA, COF/Au@HA, COF/Ir@HA, and COF/Au_0.75_/Ir_0.25_@HA NPs. a) The MTT assay of COF@HA and COF/Au@HA ([Au] fixed at 200 ppm) NPs showing nontoxicity within 3 days. b) Cytotoxicity of different iridium concentrations of COF/Ir@HA NPs within 3 days. c) Cell toxicity of different iridium concentrations of COF/Au_0.75_/Ir_0.25_@HA within 3 days. d) The visualized images of COF/Au_0.75_/Ir_0.25_@HA NPs with cells stained by Hoechst 33 342 (blue, nuclei), calcein AM (green, live cells), and EthD‐III (red, dead cells). e) The intracellular HOCl of COF/Au_0.75_/Ir_0.25_@HA NPs with cells stained by APF (green) dye. (ns: not significant. *p < 0.05, **p < 0.01, *** *p* < 0.001; *n* = 3).

The MTT assay was used to evaluate cell cytotoxicity. As shown in **Figure** **3**a, both COF and COF/Au NPs exhibited minimal cytotoxicity in hepatoma cells (HepG2). In the COF/Au system, glucose is converted to gluconic acid and H₂O₂ in the presence of O₂, which can induce starvation therapy by promoting the overproduction of toxic H₂O₂,.^[^ ^38^, ^39^, ^40^ ^]^ However, in our COF/Au system, H₂O₂ is continuously converted back to O₂ during the reaction, leading to reduced H₂O₂ levels. This decrease in H₂O₂ weakens the effectiveness of the starvation therapy, and as a result, the COF/Au system does not exhibit a significant cell starvation effect. In contrast, the cytotoxicity of COF/Ir and COF/Au₀.₇₅/Ir₀.₂₅ NPs was positively correlated with concentration and incubation time, as shown in Figure 3b,c. Cell metabolic activity decreased with increasing Ir concentration and extended incubation, suggesting that the materials require a specific duration to generate sufficient amounts of ROS and RHS to induce cytotoxicity in cancer cells. COF/Au₀.₇₅/Ir₀.₂₅ demonstrated superior cytotoxicity compared to COF/Ir, which can be attributed to the synergy of the bimetallic system. In contrast, for normal liver and breast cells (NeHepLxHT^[^ ^41^ ^]^ and MCF‐10A cells), COF/Au₀.₇₅/Ir₀.₂₅ NPs showed no significant cytotoxicity, demonstrating favorable biosafety (Figure S26a–d, Supporting Information). Additionally, an in vitro western blotting study was used to measure the cytotoxicity of COF/Au0.75/Ir0.25@HA nanoparticles and their ability to trigger apoptosis signals at the cellular level. Two types of normal cells (NeHepLxHT and MCF‐10A) and HepG2 HCC cells were treated with the material during a 3‐day co‐culture. Cell lysates were analyzed using antibodies for the apoptosis markers gamma‐H2A.x and cleaved Caspase 3. The expression levels of gamma‐H2A.x and cleaved Caspase 3 in HepG2 cells were significantly higher than in the two normal cell types, indicating a strong cytotoxic effect on the cancer cells (Figure S27, Supporting Information)

Flow cytometry results (Figure S28, Supporting Information) show the condition of hepatoma cells after 72 h of co‐culture with the materials, using thapsigargin as a positive control. To observe cytotoxicity, we used a low Ir concentration ([Ir]: 50 ppm). The flow cytometry data is consistent with the MTT assay results (Figure 3a–c), showing a similar trend in cell cytotoxicity. Both COF/Ir and COF/Au₀.₇₅/Ir₀.₂₅ induced cell apoptosis, but over a prolonged incubation period, COF/Au₀.₇₅/Ir₀.₂₅ resulted in the highest cell mortality (Control: 0.47%, COFs: 1.29%, COF/Au: 0.54%, COF/Ir: 18%, COF/Au₀.₇₅/Ir₀.₂₅: 31.35%).

### 2.4 ROS and RHS Detected in Cells Using COF@HA, COF/Au@HA, COF/Ir@HA, and COF/Au_0.75_/Ir_0.25_@HA NPs

Live and dead cell staining was performed to assess cell viability. Hoechst 33 432 (blue), calcein AM (green), and ethidium homodimer‐III (EthD‐III, red fluorescence) were used to label the nuclei, live cells, and dead cells, respectively. After 30 min of incubation, qualitative imaging was conducted (Figure 3d). COFs alone caused no cell damage, and similar to the MTT assay results, no cell death was observed in the COF/Au group. However, COF/Au₀.₇₅/Ir₀.₂₅ induced significantly more cell death than COF/Ir.

To observe RHS generation, APF fluorescence dye (green) was used to detect HOCl (Figure 3e). Neither COFs nor COF/Au showed any HOCl signal. However, the COF/Ir group displayed HOCl, while COF/Au₀.₇₅/Ir₀.₂₅ exhibited the highest HOCl generation, indicated by intense green fluorescence. We have statistically analyzed the total fluorescence signal of the confocal images, divided it by the total number of cells, and compared the results with the cell‐only control group. The quantified fluorescence images for Figure 3d,e are shown in Figure S29 (Supporting Information).

**Figure 4:**
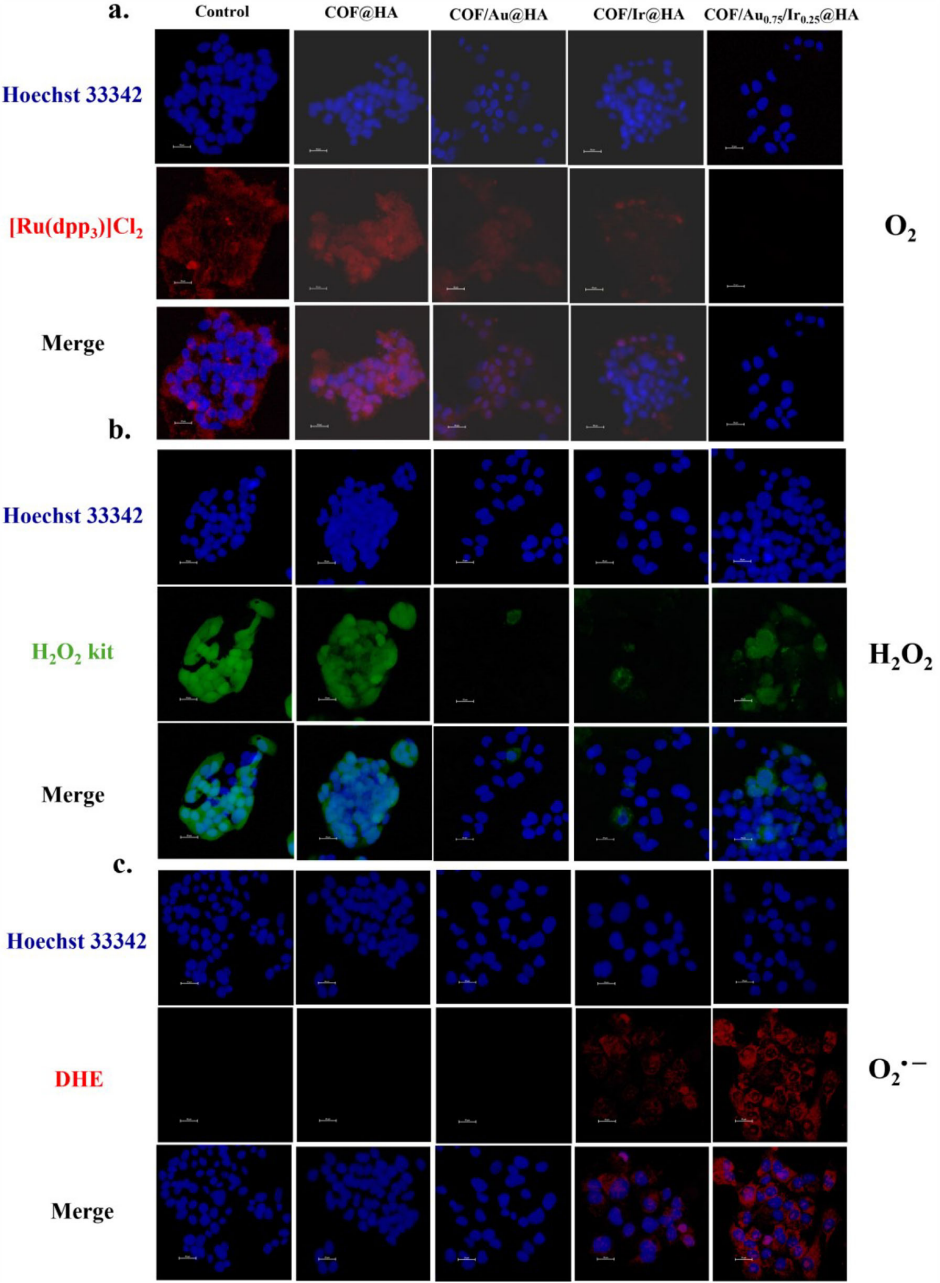
The fluorescence images of COF@HA, COF/Au@HA, COF/Ir@HA, and COF/Au_0.75_/Ir_0.25_@HA NPs for 3 days incubation with HepG2 cells. a) O_2_ emergence dyed with [Ru(dpp)_3_]Cl_2_ (red color faded when appearance of O_2_) b) The intracellular H_2_O_2_ detected by hydrogen peroxide assay kit (green). c) DHE reagent (red) used to reveal the formation of O_2_^·−^.

[Ru(dpp)₃]Cl₂ (red fluorescence) was used to track intracellular O₂ levels. Except for COFs, which showed no change, COF/Au, COF/Ir, and COF/Au₀.₇₅/Ir₀.₂₅ all demonstrated a drop in fluorescence, indicating O₂ generation (**Figure** **4**a). COF/Au₀.₇₅/Ir₀.₂₅ produced the most O₂, leading to a significant reduction in fluorescence.

A hydrogen peroxide assay kit (green fluorescence) was used to measure intracellular H₂O₂ levels. In the control group (without materials), green fluorescence indicated the presence of intracellular H₂O₂ (Figure 4b). COFs did not affect H₂O₂ levels compared to the control group. In contrast, the luminescence in the COF/Au, COF/Ir, and COF/Au₀.₇₅/Ir₀.₂₅ groups decreased effectively. Interestingly, COF/Au₀.₇₅/Ir₀.₂₅ did not show the highest H₂O₂ consumption. As previously noted, COF/Au₀.₇₅/Ir₀.₂₅ has the greatest capacity to participate in a cyclic reaction. Figure 2c shows that COF/Au₀.₇₅/Ir₀.₂₅ generates the most O₂ through the glucose + O₂ → H₂O₂ → O₂ cycle, indicating a fast O₂ production rate. The bimetallic synergistic effect likely accelerates glucose oxidation by Au to produce H₂O₂. This O₂ is recycled in the intracellular cycle, reacting with glucose to form more H₂O₂, resulting in a higher H₂O₂ production rate than consumption, which is why H₂O₂ is still observed in cell imaging. In contrast, COF/Au oxidizes glucose to produce H₂O₂, which is subsequently decomposed into O₂, while COF/Ir reacts with endogenous H₂O₂, decomposing it into O₂ and O₂^•─^ or reacting with Cl⁻ to form HOCl. To avoid misunderstanding, it is noted that Figure 2g is not performed from cell studies but rather involves manually adding H₂O₂.

ROS measurements used dihydroethidium (DHE, red fluorescence) to detect O₂^•─^. Consistent with the ESR results (Figure 2f), COFs and COF/Au did not generate O₂^•─^, whereas COF/Ir and COF/Au₀.₇₅/Ir₀.₂₅ did (Figure 4c). COF/Au₀.₇₅/Ir₀.₂₅ produced the most O₂^•─^ through the glucose + O₂ → H₂O₂ → O₂^•─^ pathway. We have quantified cell imaging data for the generated reactive species by taking fluorescence intensity to compare with the control group. The quantified fluorescence images for Figure 4a–c are provided in Figure S30 (Supporting Information).

Finally, to assess the material's potential impact on the bloodstream before animal studies, we measured the hemolysis rate by incubating the materials with 2% red blood cells. Results indicated no hemolysis in any group (Figure S31, Supporting Information).

### 2.5 Density Functional Theory (DFT) Calculation of COF Containing Au and Ir Structures

**Figure 5:**
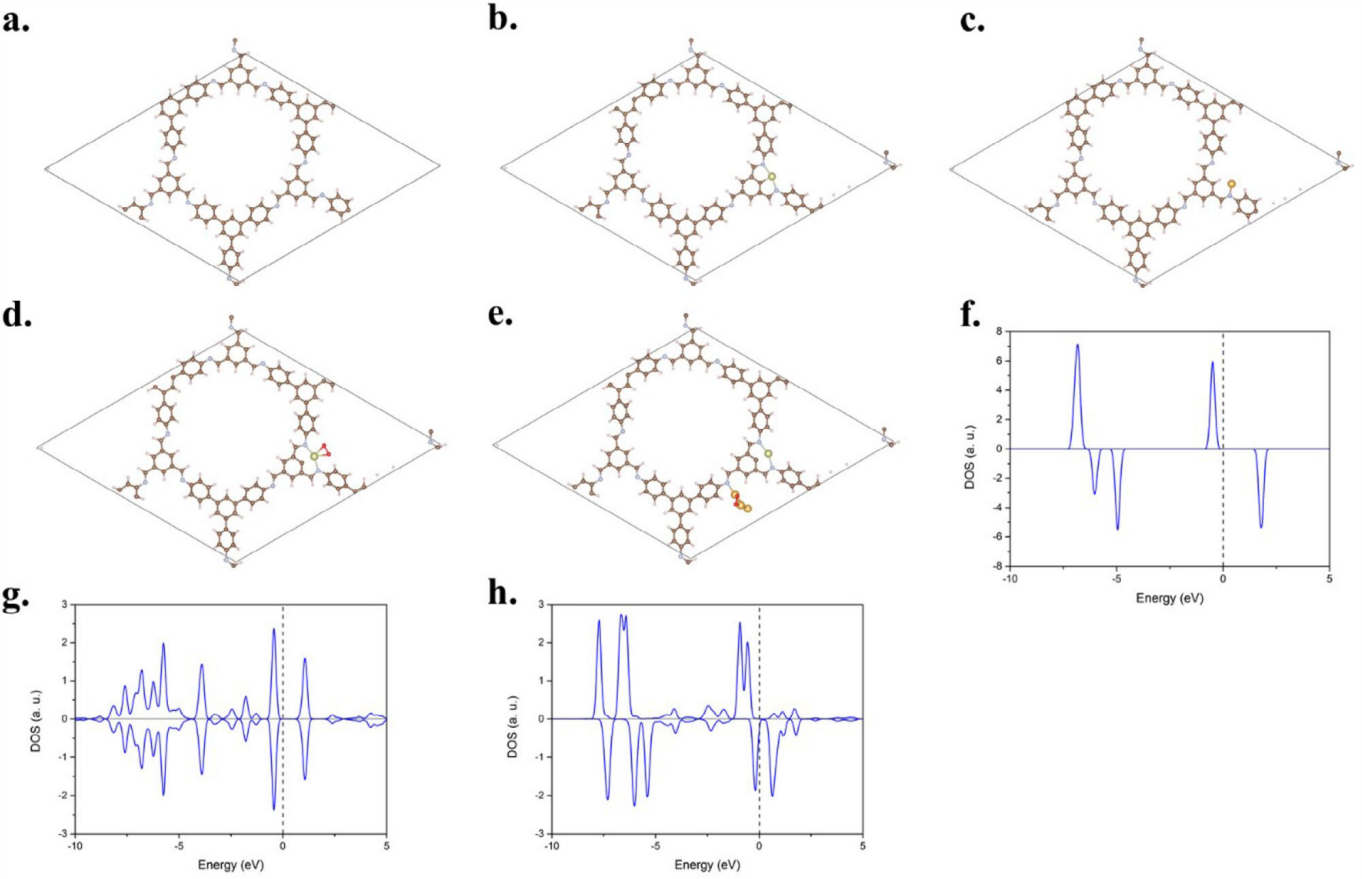
DFT optimized structures of a) COF. b) Ir atom on the bidentate nitrogen chelate site of COF. c) Au atom on the monodentate nitrogen site of COF. d) The adsorption structure of the O_2_ molecule on the COF/Ir. e) the adsorption structure of the O_2_ molecule on the COF/Au_3_. Calculated projected density of states (PDOS) of f) free O_2_ molecule. g) the adsorption of the O_2_ molecule on the COF/Ir. h) the adsorption of O_2_ molecule on the COF/Au_3_. The dashed line in the PDOS diagrams represents the Fermi level.

To further understand the catalytic property, we conducted density functional theory calculations. The optimized structure of the TAPB‐BTCA‐COF (C_99_H_63_N_9_) is drawn in **Figure** **5**a, in which the optimized lattice parameters of the COF are *a *= 32.48 Å and *b* = 31.66 Å. We first calculate the binding energy of Au and Ir single atoms on the COF. Possible adsorption sites include η^1^‐CN (carbon of the nitrile group), η^1^‐C_6_H_5_ (benzene), η^6^‐C_6_H_5_ (benzene), monodentate nitrogen, and bidentate nitrogen chelate sites. The calculated binding energy of Ir atom on η^1^‐C_6_H_5_, η^6^‐C_6_H_5_, monodentate nitrogen, and bidentate nitrogen chelate sites is −2.65, −2.05, −2.47, and −5.18 eV, respectively, as shown in Figure 5b,c and Figure S32 (Supporting Information). Besides, the calculated binding energy of the Au atom on η^1^‐CN, η^6^‐C_6_H_5_, monodentate nitrogen, and bidentate nitrogen chelate sites is −0.41, −0.15, −0.53, and −0.42 eV, respectively. These results reveal that the Ir single atom possesses an extremely strong binding ability to the COF on the bidentate nitrogen chelate site. In contrast, the Au single atom tends to be adsorbed on the monodentate nitrogen site. Both the Ir and Au atoms can form a single atom catalyst on the COF.

In addition, from a molecular structural perspective, each COF framework has only three positions capable of generating such bidentate coordination sites. As the chemical formula of the TAPB‐BTCA‐COF is C_99_H_63_N_9_, when only three Ir atoms are adsorbed on the bidentate nitrogen chelate sites, the theoretical molar ratio is ≈0.295. Since the number of bidentate nitrogen chelate sites available on the COF for forming Ir single atoms is limited, an excessive amount of Ir leads to the formation of clusters, reducing activity. Based on atomic ratios, when all available bidentate coordination sites are occupied by Ir, the theoretical molar ratio closely matches the molar ratio calculated from our experimental conditions using 20 µL of IrCl_3_. The experimental results also show the optimal effect when 20 µL of IrCl_3_ is used, forming the COF/Au₀.₇₅/Ir₀.₂₅ group.

We further calculated the adsorption of the O_2_ molecule on the COF/Au_0.75_/Ir_0.25_ group. Because the Au/Ir ratio in the COF/Au_0.75_/Ir_0.25_ group is 3:1, we adopted three Au atoms and one Ir atom to be co‐adsorbed on the COF. The adsorption energy of the O_2_ molecule on the Ir atom and the Au_3_ is −1.51 and −1.19 eV, respectively, as shown in Figure 5d,e. Furthermore, the bond length of O─O bond on Ir atom and Au_3_ is elongated to 1.42 and 1.3 Å, respectively. We also calculated the projected density of states (PDOS) of the O_2_ molecule on the Ir atom and the Au_3_, as shown in Figure 5f–h. It shows no spin splitting of the O_2_ molecule on the Ir atom, but the spin splitting still exists for the O_2_ molecule on the Au_3_. This result suggests that the O_2_ molecule on the Ir atom becomes O₂•⁻ while the O_2_ molecule does not gain more electrons to form the O₂•⁻ from the Au_3_. Hence, DFT calculations indicate that only the Ir single atom can produce the O₂•⁻, in good agreement with the experimental observations.

### 2.6 In Vivo Orthotopic Hepatocellular Carcinoma Model Studies in COF/Au_0.75_/Ir_0.25_ NPs

**Figure 6:**
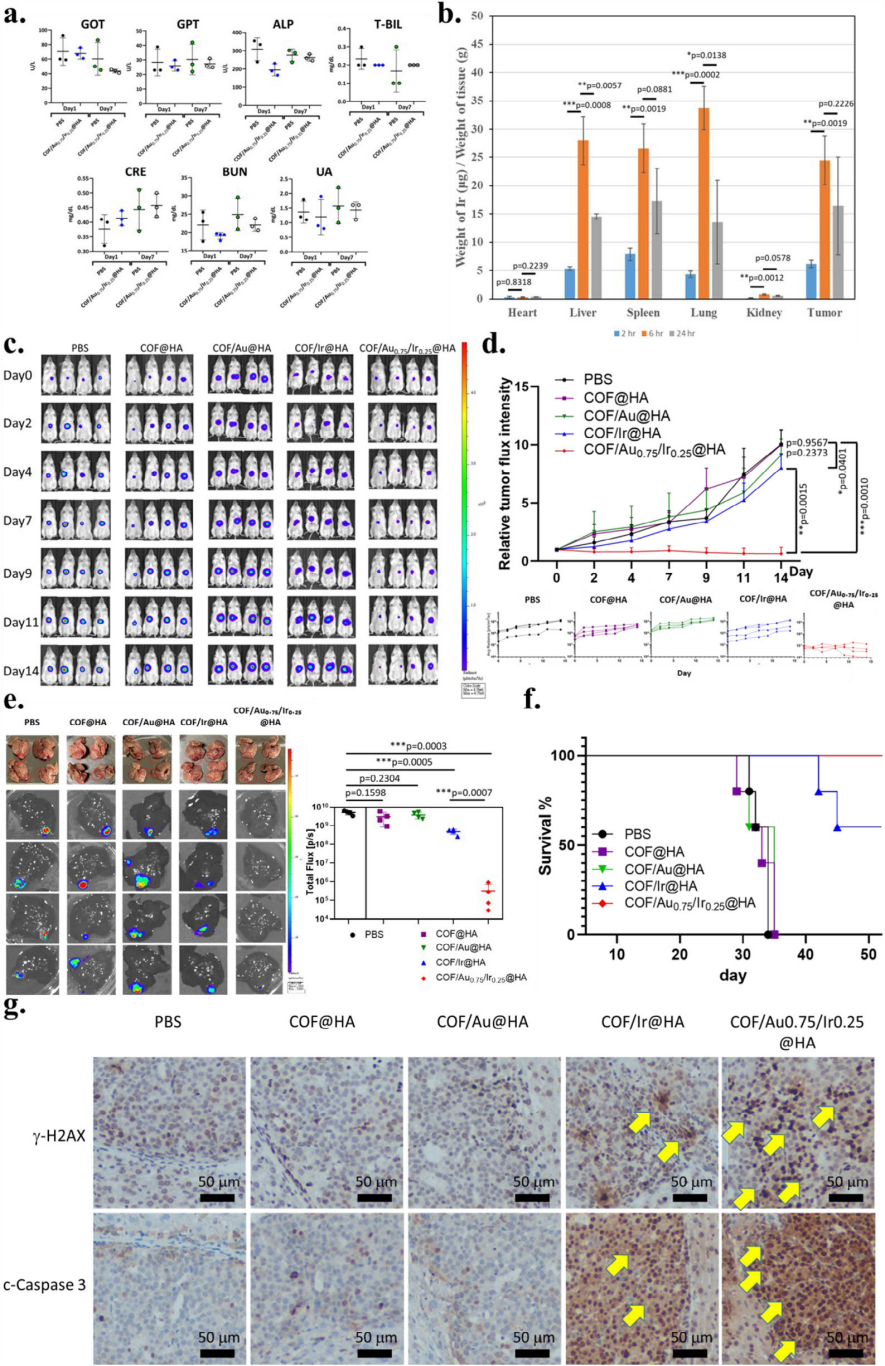
In vivo bio‐safety and anti‐tumor activity of the mice with HepG2‐Red‐FLuc orthotopic tumors. a) The blood biochemical analysis was determined on post‐injection day 1 and 7 of C57BL/6 mice with PBS and COF/Au_0.75_/Ir_0.25_@HA NPs through intravenous injection (ALP: alkaline phosphatase, T‐BIL: total bilirubin, CRE: creatinine, BUN: blood urea nitrogen, UA: uric acid, *n* = 3). The error bars in represented mean ± SEM. b) The biodistribution was determined by the Ir concentration collected from treated mice (*n* = 3). c) The IVIS bioluminescence of hepatocellular carcinoma in each treatment group, and the individual luminescence was showed at the under panel. d) Tumor volume growth curves of mice after various treatments (pre‐ to post‐14 days, *n* = 4). e) Ex vivo images of livers extracted from mice after 14 days of treatment. (*n* = 4). Red dashed circles indicate the tumor regions. The IVIS bioluminescence of livers with hepatocellular carcinoma in each treatment group was determined (*n* = 4). f) The survival rate of orthotopic hepatocellular carcinoma xenograft mice treated with PBS, COF@HA, COF/Au@HA, COF/Ir@HA, and COF/Au_0.75_/Ir_0.25_@HA was evaluated (*n* = 5 per group). g) The expression of phosphor‐histone H2AX and cleaved caspase‐3 within hepatocellular carcinoma from each treatment group mouse by IHC staining (scale bar, 50 µm). All images are representative of at least three independent experiments. Data are mean ± s.d. Statistical analysis was performed using a two‐tailed Student's *t*‐test for comparisons.

While the HA‐modified COF/Au₀.₇₅/Ir₀.₂₅ remains stable with good biocompatibility in cell culture, in vivo systemic toxicity remains to be confirmed before conducting the antitumor efficacy experiment. C57BL/6 mice were intravenously administered with COF/Au₀.₇₅/Ir₀.₂₅@HA (in sterilized PBS). Seven‐day toxicity studies reveal no significant change in body weight, blood biochemical index (for liver and kidney functions), or organ histology of heart, lung, liver, spleen, and kidney, compared to the control group, indicating excellent biocompatibility. (**Figure** **6**a; Figures S33 and S34, Supporting Information). Biodistribution studies demonstrate preferential tumor and lymphatic‐related organs (lung and spleen)^[^ ^7^ ^]^ accumulation of the COF/Au₀.₇₅/Ir₀.₂₅@HA. Interestingly, most of the organ accumulations reveal rapid clearance post‐6 to ‐24 h i.v. injection, however, the tumor shows especially enhanced retention in orthotopic hepatocellular carcinoma (Figure 6b).

To evaluate antitumor efficacy, HepG2‐Red‐FLuc orthotopic hepatocellular carcinoma xenograft mice were treated with various formulations, according to the consideration of the 3Rs (replacement, reduction, and refinement) under the approval of the Institutional Animal Care and Use Committee of National Cheng Kung University. The tumor‐bearing mice were divided into five treatment cohorts: PBS (control), COF@HA, COF/Au@HA, COF/Ir@HA, and COF/Au₀.₇₅/Ir₀.₂₅@HA. Except for the PBS group, all cohorts received intravenous nanoparticle (NP) treatments with two doses of 100 µg mouse^−1^ administered through the tail vein (one dose per week over two weeks). Tumor‐suppressing efficacy was assessed by measuring bioluminescence at tumor sites using an in vivo imaging system (IVIS). As shown in Figure 6c,d, bioluminescence signals from hepatocellular carcinoma increase significantly in the PBS, COF@HA, and COF/Au@HA groups. In contrast, the COF/Au_0.75_/Ir_0.25_@HA group demonstrates substantial suppression of bioluminescence signals. The COF/Ir@HA group shows a certain degree of suppression of bioluminescence compared to PBS, aligning with the cytotoxicity data. Ex vivo bioluminescence images of dissected livers corroborated the IVIS findings, revealing consistency with the observed tumor‐suppressing effects across treatment groups. Notably, liver tissue imaging provided a clearer assessment of tumor suppression, highlighting the superior efficacy of the COF/Au_0.75_/Ir_0.25_@HA treatment (Figure 6e). The administration of COF/Au_0.75_/Ir_0.25_@HA markedly extended the survival of orthotopic HCC xenograft mice, with no mortality recorded up to 50 days post‐treatment (Figure 6f).

Histological analysis reveals morphological changes in the tumors. Haematoxylin and eosin (H&E) staining shows that COF/Au_0.75_/Ir_0.25_@HA treatment induced more extensive tumor necrosis compared to other groups (Figure S35, Figure 6a). The presence of necrotic and fibrotic tumor remnants indicates robust therapeutic efficacy. Additionally, immunohistochemistry analysis of phospho‐histone H2A.X and cleaved caspase‐3 proteins demonstrates significant DNA damage and apoptosis in tumor sections following COF/Au_0.75_/Ir_0.25_@HA treatment, as evidenced by prominent brown‐stained areas (Figure 6g). Taken together, these results indicate pronounced abnormalities in tumor sections, marked DNA damage, and extensive cell death, underscoring the superior anticancer efficacy of COF/Au_0.75_/Ir_0.25_@HA in vivo. This study highlights its potential as a promising therapeutic agent for hepatocellular carcinoma, offering a new nanoplatform for clinical applications.

## 3 Conclusion

This study introduces a novel cancer treatment approach using dual metal atomic sites on COFs, engineered through a galvanic reaction between Au and Ir. By precisely controlling the Au: Ir ratio, we optimized the catalytic properties of the NPs, enabling synergistic glucose oxidation and the generation of ROS and RHS within cancer cells. The bimetallic system efficiently leverages endogenous cancer cell metabolites like glucose, hydrogen peroxide (H₂O₂), and chloride ions (Cl⁻) to produce superoxide anion (O₂⁻) and hypochlorous acid (HOCl), while recycling H₂O₂ back into oxygen. This sustained ROS and RHS production selectively targets cancer cells without affecting normal cells. As the first application of dual metal atomic sites on COFs for cancer therapy, this approach demonstrates the potential of bimetallic catalysis for highly selective and efficient cancer treatments.

## 4 Experimental Section

### Materials

All regents were analytical purity and used without further purification. 1,3,5‐Tris(4‐aminophenyl) benzene (TAPB; C_24_H_21_N_3_, 97%), 1,3,5‐Triformylbenzene (BTCA; C_9_H_6_O_3_, 97%), Hydrogen tetrachloroaurate (III) trihydrate (HAuCl_4_, 99.99%), Iron (III) chloride (FeCl_3_, 98%), Disodium terephthalate (TPA, C_8_H_4_Na_2_O_4_, ≥99%) were bought from Thermo Fisher. Polyvinylpyrrolidone (PVP; (C_6_H_9_NO)_n_, M.W. = 55 000), Iridium (III) chloride hydrate (IrCl_3_, 99.9%), Sodium borohydride (NaBH_4_, 99%), Hyaluronic acid sodium salt from rooster comb (HA; (C_14_H_21_NaNO_11_)_n_), 3‐(4,5‐dimethylthiazol‐2‐yl)‐2,5‐diphenyltetrazolium bromide (MTT, C_18_H_16_BrN_5_S, 97.5%), Hydroxylamine solution (NH_2_OH, 50%), Trichloroacetic acid (CCl_3_COOH, ≥99%), *D*‐(+)‐Glucose (C_6_H_12_O_6_, ≥99.5%), D‐Gluconic acid sodium salt (C_6_H_11_NaO_7_, ≥99%), Dihydroethidium (DHE, C_21_H_21_N_3_, ≥95%), Annexin V‐FITC Apoptosis Detection Kit were obtained from Sigma Aldrich. Acetonitrile (CH_3_CN, >99.9%), Ethanol (C_2_H_5_OH, ≥99.5%) were purchased from ECHO. Acetic acid (CH_3_COOH, 90%) was bought from CAMEO Chemicals. Water was obtained by using a Millipore direct‐Q deionized water system throughout all studies. Minimum essential medium (MEM), High‐glucose Dulbecco's modified Eagle's medium (DMEM), MEM, Dulbecco's modified Eagle's medium/Ham's F‐12 Mixture (DMEM/F‐12), and FBS were purchased from Gibco. Penicillin–streptomycin were obtained from Caisson. Hoechst 33 342, calcein AM, and EthD‐III were procured from Invitrogen. Hydrogen peroxide (H_2_O_2_, 30.0%) was bought from Honeywell Fluka. Methanol (CH_3_OH, ≥99.9%) was obtained from Macron Fine Chemicals. Aminophenyl fluorescein solution (APF, C_26_H_17_NO_5_, 98%) was acquired from Life Technologies. Tris(4,7‐diphenyl‐1,10‐phenanthroline) ruthenium(II) dichloride complex ([Ru(ddp)_3_]Cl_2_, C_72_H_48_Cl_2_N_6_Ru) were purchased from Alfa Aesar. Antibiotic‐Antimycotic was acquired from Simply Biologics. Hydrogen Peroxide Assay Kit (Cell‐based) was obtained from Abcam. Trimethylamine (Me_3_N, 30%) was bought from SHOWA. Ethylenediaminetetraacetic acid (EDTA, C_10_H_16_N_2_O_8_, 99%) was purchased from Lancaster.

### Preparation of Covalent Organic Frameworks (COFs)

Dissolved 20 mg of TAPB and 10 mg of BTCA in 20 mL of CH_3_CN. The mixture was stirred at room temperature until uniform, then added 1 mL of PVP (0.1 g mL^−1^ in CH_3_CN) and 100 µL of CH_3_COOH. Continued stirring at room temperature for 24 h. Afterward, centrifuged at 15 000 rpm for 10 min to collect the precipitate. The precipitate was washed three times using methanol, 50%/50% methanol/ethanol (volume/volume), and ethanol, respectively. Finally, dispersed the COFs in ethanol for further use.

### Preparation of Iridium Doped COF (COF/Ir)

PVP (20 mg) was mixed with 4 mL of COFs in a flask. Next, diluted 0 (COFs only), 5, 10, 20, 50, and 100 µL of 0.1 m IrCl_3_ in 10 mL of deionized water, and added it to the aforementioned flask. Stirred continuously at room temperature for 12 h. A condenser was attached to the flask containing the mixture and heated it in an oil bath at 100 °C for 6 h. After cooling, centrifuged at 15 000 rpm for 10 min to collect the precipitate. The precipitate was washed three times with ethanol, and finally dispersed the COF/Ir in ethanol for further use.

### Preparation of Gold Doped COF (COF/Au)

PVP (50 mg), 10 mL of COFs, and 10 mL of deionized water were mixed in a flask. Stirred continuously at room temperature for 12 h. A condenser was attached to the flask containing the mixture and heated it in an oil bath at 100 °C for 6 h. After cooling, centrifuged at 15 000 rpm for 10 min to collect the precipitate. The precipitate was washed three times with ethanol, and finally stored the COF in ethanol. Next, 2 mL of COFs was taken and stirred it with different volumes (0.5, 0.75, 1, 5, and 10 µL) of 50 mm HAuCl_4_ in a light‐protected environment for 30 min. Centrifuged at 15 000 rpm for 10 min to collect the precipitate, removing any unreacted HAuCl_4_. After redispersing in ethanol, added the corresponding volumes of 50 mm NaBH_4_ (2.5, 3.75, 5, 25, and 50 µL) and stirred for 1 min. Centrifuged at 15 000 rpm for 10 min to collect the precipitate. The precipitate was washed three times with ethanol, and finally dispersed the COF/Au in ethanol for further use.

### Preparation of Gold/Iridium Doped COF (COF/Au_x_/Ir_1‐x_)

COF/Ir (2 mL) was mixed with different volumes (5, 10, 20, 50, and 100 µL) of 50 mm HAuCl_4_, and stirred in a light‐protected environment for 30 min. Centrifuged at 10 000 rpm for 10 min to collect the precipitate, then washed it three times with ethanol. Finally, dispersed the COF/Au_x_/Ir_1‐x_ in ethanol for further use.

### Preparation of COF/Au_x_/Ir_1‐x_@HA

First, 1 mg of hyaluronic acid sodium salt was dissolved in 1 mL of ice‐cold deionized water. Once dissolved, 1 mL of ethanol was added and mixed. Stirred the prepared solution with 2 mL of COF/Au_x_/Ir_1‐x_ in an ice bath for 10 min. Centrifuged at 10 000 rpm for 10 min to collect the precipitate, then washed it three times with deionized water. Finally, dispersed the COF/Au_x_/Ir_1‐x_@HA in water and stored it at 4 °C for future experiments. Other COF@HA (0 µL of 100 mm IrCl_3(aq)_ when synthesis of COF/Ir was chosen), COF/Ir@HA (20 µL of 100 mm IrCl_3(aq)_ when synthesis of COF/Ir was chosen), and COF/Au@HA (1 µL of 50 mm HAuCl_4(aq)_ when synthesis of COF/Au was chosen) samples were modified using the same method.

### Characterization of Atomic Environment

X‐ray absorption spectroscopy, including X‐ray absorption near‐edge structure (XANES) and extended X‐ray absorption fine structure (EXAFS) for the Au foil, Ir powder, COF/Au, and COF/Au_0.75_/Ir_0.25_ samples were performed in fluorescence mode using a Lytle detector for the Au and Ir L₃‐edges at beamline TLS 01C1 at the National Synchrotron Radiation Research Center (NSRRC) Taiwan. The TLS 01C1 beamline used a double Si(111) crystal monochromator from a 5.0 Tesla magnet as a wavelength‐shifted light source for the 1.5 GeV synchrotron ring and operated at 360 mA. Behind the monochromator was a Pt‐coated focusing mirror. Due to the design of the beamline optics, only the XANES spectrum at the Ir *L*₃‐edge and the EXAFS spectrum at the Au *L*_3_‐edge were collected. The spectra were processed by subtracting the pre‐edge baseline and normalizing the post‐edge using Athena software.^[^ ^42^ ^]^ EXAFS analysis for the Au *L*₃‐edge was carried out by Fourier transforming k^3^‐weighted EXAFS oscillations to identify the contributions of different atomic shells. The subsequent fitting was performed using Artemis software. The powder X‐ray diffraction patterns were collected at the TLS 01C2 beamline using a mar345 imaging plate detector.

### Evaluation of Gluconic Acid Generation upon Glucose Existence

In the procedure of gluconic acid generation, Au could catalyze glucose + O_2_ into gluconic acid and H_2_O_2_. Gluconic acid could react with NH_2_OH and FeCl_3_ forming into red hydroxamate‐Fe^3^⁺ complex which could be measured the absorbance at 505 nm and then quantified the concentration of gluconic acid. In brief, 1 mL of COFs (weighing concentration: 10 ppm), COF/Au with different volume of 50 mm HAuCl_4(aq)_ (Au concentration: 10 ppm), COF/Ir (Ir concentration: 10 ppm), different ratios of COF/Au_x_/Ir_1‐x_ (Au concentration: 10 ppm), and COF/Au_0.75_/Ir_0.25_@HA (Au concentration: 10 ppm) NPs in 0.4 mm glucose solution, followed by 24 h incubation at 37 °C. After centrifugation, 100 µL of the supernatant was mixed with 250 µL of solution 1 (5 mm EDTA + 0.15 mm Me_3_N). After that, the solution was added with 25 µL of solution 2 (3 m NH_2_OH), and the mixture was proceeded to react for 15 min. Finally, 125 µL of solution 3 (1 m HCl + 0.1 m FeCl_3_ + 0.25 m CCl_3_COOH) was added to the mixture, and was allowed to react for 5 min, and then was measured at an absorbance wavelength of 505 nm by using an ELISA reader.

### Quantitation of Hydrogen Peroxide Generation under Glucose Existence

The H_2_O_2_ assay kit (abcam) used the AbGreen indicator to quantify the concentration of H_2_O_2_. First, the AbGreen indicator was diluted in 50 µL of DMSO and then added it into 5 mL of Assay buffer. For the standard calibration curve, the 1 mL solutions containing 100 µL of AbGreen solution with different concentrations of H_2_O_2_ (0, 10, 20, 50, and 100 °m) for 30 min reaction. The control group consisted of 100 µL of AbGreen solution and 900 µL of PBS, which the mixture included 0.4 mm glucose. For each sample, 1 mL of COFs (weighing concentration: 10 ppm), COF/Au (Au concentration: 10 ppm), COF/Ir (Ir concentration: 10 ppm), different ratios of COF/Au_x_/Ir_1‐x_ (Ir concentration: 10 ppm), and COF/Au_0.75_/Ir_0.25_@HA (Ir concentration: 10 ppm) NPs in 0.4 mm glucose and contained 100 µL of AbGreen solution. After reacting for 30 min, the solution was centrifuged at 15 000 rpm for 10 min. Thereafter, the supernatant was measured its signal (ex/em:490/515 nm) by fluorescence spectrometer. The relevant fluorescence intensity was proportional to the quantity of H_2_O_2_.

### Evaluation of Oxygen Generation

Before O_2_ measurements, all solvents used for preparation were purged with N_2(g)_ to minimize O_2_. The [Ru(ddp)_3_]Cl_2_ was used as an oxygen probe, which was dissolved in ethanol (400 °m). The control group consisted of 100 µL of [Ru(ddp)_3_]Cl_2_ solution and 900 µL of deoxygenation H_2_O. For each sample, 1 mL of COFs (weighing concentration: 10 ppm), COF/Au (Au concentration: 10 ppm), COF/Ir (Ir concentration: 10 ppm), different ratios of COF/Au_x_/Ir_1‐x_ (Ir concentration: 10 ppm), and COF/Au_0.75_/Ir_0.25_@HA (Ir concentration: 10 ppm) NPs, respectively, was in 100 °m H_2_O_2_, 0.4 mm glucose, or H_2_O_2_ + glucose mixture and also contained 40 °m of [Ru(ddp)_3_]Cl_2_. After reaction for 30 min under dark, the solution was centrifuged at 15 000 rpm for 10 min. Afterward, the supernatant was analyzed for its luminescence intensity (ex/em: 455/618 nm) using a fluorescence spectrometer. The relative ratio of fluorescence intensity was inversely proportional to the generation of oxygen.

### Evaluation of Hydroxyl Radical Generation under Endogenous Hydrogen Peroxide

The TPA solution was used to detect ·OH and was dissolved in PBS (1 mg mL^−1^). The control group was composed of 100 µL of TPA solution and 900 µL of H_2_O. For every sample, 1 mL of COFs (weighing concentration: 10 ppm), COF/Au (Au concentration: 10 ppm), COF/Ir with different volumes of 100 mm IrCl_3(aq)_ (Ir concentration: 10 ppm), COF/Au_0.75_/Ir_0.25_ (Ir concentration: 10 ppm) NPs was respectively in 100 °m H_2_O_2_ and contained 100 µL of TPA solution. After reacting for 0 and 30 min, the solution was analyzed for its emission intensity (ex/em: 310/425 nm) using a fluorescence spectrometer, separately. The corresponding ratio of intensity (*I*_30 min_/*I*_0 min_) could reflect the amount of ·OH.

### Evaluation of Hypochlorous Acid Generation

The APF was used to detect HOCl and was diluted in PBS (10 °m). The control group was composed of 100 µL of APF solution and 900 µL of PBS. For every sample, 1 mL of COFs (weighing concentration: 10 ppm), COF/Au (Au concentration: 10 ppm), COF/Ir with different volumes of 100 mm IrCl_3(aq)_ (Ir concentration: 10 ppm), different ratios of COF/Au_x_/Ir_1‐x_ (Ir concentration: 10 ppm), and COF/Au_0.75_/Ir_0.25_@HA (Ir concentration: 10 ppm) NPs in 100 °m H_2_O_2_ and contained 100 µL of APF solution. After reacting for 0 and 30 min, the solution was analyzed for its light emission intensity (ex/em: 490/515 nm) using a fluorescence spectrometer, respectively. The associated ratio of intensity (*I*_30 min_/*I*_0 min_) could reflect the amount of HOCl.

### Evaluation of Superoxide Anions by ESR

O_2_^•‐^ was detected by CMH and quantified by ESR. The control group was made up of 100 µL of 0.1 m CMH in PBS and 100 µL of PBS. For each category, 100 µL of COFs (concentration: 10 ppm), COF/Au (Au concentration: 10 ppm), COF/Ir with different volumes of 100 mm IrCl_3(aq)_ (Ir concentration: 10 ppm), different ratios of COF/Au_x_/Ir_1‐x_ (Ir concentration: 10 ppm), and COF/Au_0.75_/Ir_0.25_@HA (Ir concentration: 10 ppm) NPs, respectively, were in 100 °m H_2_O_2_, 0.4 mm glucose or H_2_O_2_ + glucose mixture, and were mixed with 100 µL of 0.1 m CMH. After reacting for 0 and 30 min, the solution was analyzed by an ESR spectrometer (Bruker E580, Germany).

### Quantitation of Hydrogen Peroxide Consumption under Endogenous Hydrogen Peroxide

The H_2_O_2_ assay kit (abcam) used the AbGreen indicator to quantify the concentration of H_2_O_2_. First, the AbGreen indicator was diluted in 50 µL of DMSO and then added it into 5 mL of Assay buffer (AbGreen solution). For the standard calibration curve, the 1 mL solutions containing 100 µL of AbGreen solution with different concentrations of H_2_O_2_ (0, 10, 20, 50, and 100 °m) for a 30 min reaction. The control group consisted of 100 µL of AbGreen solution and 900 µL of PBS which the mixture included 100 °m H_2_O_2_. For each sample, 1 mL of COFs (weighing concentration: 10 ppm), COF/Au (Au concentration: 10 ppm), COF/Ir (Ir concentration: 10 ppm), different ratios of COF/Au_x_/Ir_1‐x_ (Ir concentration: 10 ppm), and COF/Au_0.75_/Ir_0.25_@HA (Ir concentration: 10 ppm) NPs were respectively in 100 °m H_2_O_2_ and contained 100 µL of AbGreen solution. After reacting for 30 min, the solution was centrifuged at 15 000 rpm for 10 min. Thereafter, the supernatant was measured its glow (ex/em: 490/515 nm) by fluorescence spectrometer. The relevant fluorescence intensity was proportional to the quantity of H_2_O_2_.

### Stability

The COF/Au_0.75_/Ir_0.25_@HA NPs (Ir concentration: 10 ppm) were separately dispersed in 1 mL of H_2_O, PBS (pH 7), PBS (pH 5), and medium. After incubation for 0, 1, 3, 5, and 7 days, the solution was washed with H_2_O twice and then observed changes of morphology through TEM.

### Cell culture

Hep G2 (Human liver cancer cell line) cells were cultured in MEM containing 10% FBS and 1% Antibiotic–Antimycotic in the incubator at 37 °C and 5% CO_2_. NeHepLxHT (Human hepatocyte cell line) cells were cultured in DMEM‐F12‐high glucose containing 100 nm dexamethasone, 0.1% ITS premix, human EGF (20 ng mL^−1^), ciprofloxacin (5 mg mL^−1^), 10% FBS, and 1% penicillin–streptomycin in the incubator at 37 °C and 5% CO_2_. MCF‐10A (Human normal breast epithelial cell line) cells were cultured in DMEM containing 10% FBS and 1% Antibiotic–Antimycotic in the incubator at 37 °C and 5% CO_2_.

### Cytotoxicity Assay

Hepatoma cells were seeded in 96‐well plates (10 000 cells well^−1^) and incubated for 24 h. After the medium was removed, fresh medium solution was added with different kinds of samples (COF@HA (weighing concentration: 100 ppm), COF/Au@HA (Au concentration: 200 ppm), COF/Ir@HA (Ir concentration: 50, 100, and 200 ppm), and COF/Au_0.75_/Ir_0.25_@HA (Ir concentration: 50, 100, and 200 ppm) NPs). After an additional co‐cultivation for 24, 48, and 72 h, he culture medium was removed and then washed the cells twice with PBS. Afterward, treated cells were added with MTT dye in fresh medium (0.5 mg mL^−1^) and cultured for another 4 h. Once again, the medium was replaced with DMSO to solubilize the purple product (formazan). Finally, an ELISA reader was used to measure the absorbance wavelength at 540 nm. The corresponding absorbance values could be related to cytotoxicity. Hepatocyte (NeHepLxHT) and breast epithelial (M10) cells were separately seeded in 96‐well plates (10 000 cells well^−1^) and incubated for 24 h. After the medium was removed, the fresh medium solution was added with COF/Au_0.75_/Ir_0.25_@HA (Ir fixed concentration: 200 ppm) NPs. After an additional co‐cultivation for 24, 48, and 72 h, remove the culture medium and then wash the cells twice with PBS. Afterward, treated cells were added with MTT dye in fresh medium (0.5 mg mL^−1^) and cultured for another 4 h. Once again, the medium was replaced with DMSO to solubilize the purple product (formazan). Finally, an ELISA reader was used to measure the absorbance wavelength at 540 nm. The corresponding absorbance values could be related to cytotoxicity.

### Cell Imaging in Observing Cytotoxicity

Live and dead showed the visualization of cell cytotoxicity, Hep G2 cells (10 000 cells well^−1^) were inoculated into the 8‐well chamber and cultured for 72 h. Then varied samples was mixed with medium (COF@HA (weighing concentration: 100 ppm), COF/Au@HA (Au concentration: 200 ppm), COF/Ir@HA (Ir concentration: 200 ppm), and COF/Au_0.75_/Ir_0.25_@HA (Ir concentration: 200 ppm) NPs) and displaced the original medium. Subsequently, cells were rinsed twice with PBS and stained with 2 °m of Hoechst 33 342 (nuclei), 1 °m of calcein AM (live cells), and 1 °m of EthD‐III (dead cells) for 30 min. Eventually, the cells were gently washed twice and observed by laser scanning confocal microscope. NeHepLxHT and M10 cells (10 000 cells well^−1^) were individually inoculated into the 8‐well chamber and cultured for 72 h. Then COF/Au_0.75_/Ir_0.25_@HA (Ir fixed concentration: 200 ppm) NPs were mixed with the medium and displaced the original medium. Subsequently, cells were rinsed twice with PBS and stained with 2 °m of Hoechst 33 342 (nuclei), 1 °m of calcein AM (live cells), and 1 °m of EthD‐III (dead cells) for 30 min. Eventually, the cells were gently washed twice and observed by laser scanning confocal microscope.

### Cell Imaging in Observing Intracellular ROS and RHS Generation

The HepG2 cells were seeded in 8‐well chamber (10 000 cells well^−1^) and cultured for 24 h. After that, different samples were mixed with medium (COF@HA (weighing concentration: 50 ppm), COF/Au@HA (Au concentration: 50 ppm), COF/Ir@HA (Ir concentration: 50 ppm), and COF/Au_0.75_/Ir_0.25_@HA (Ir concentration: 50 ppm) NPs) and exchanged the old medium. After 72 h of co‐cultivation, the cells were rinsed with PBS twice and then treated with APF dye (10 °m), [Ru(dpp)_3_]Cl_2_ (4 °m), H_2_O_2_ assay kit (100 µL of diluted aforementioned AbGree solution 250‐fold in medium), and DHE (5 °m) for 30 min, individually, to examine intracellular HOCl, O_2_, H_2_O_2_, and O_2_^•‐^.

### Flow Cytometry

The HepG2 cells were seeded in a 6‐cm culture dish (2 × 10^5^ cells well^−1^) and incubated 24 h. The cells were treated with different groups of materials mixed with medium (COFs@HA (weighing concentration: 50 ppm), COF/Au@HA (Au concentration: 50 ppm), COF/Ir@HA (Ir concentration: 50 ppm), and COF/Au_0.75_/Ir_0.25_@HA (Ir concentration: 50 ppm) nanoparticles). Additionally, the control cells were applied to this experiment (2 °m thapsigargin as positive control and medium only as negative control). After 72 h of co‐cultivation, the cells were washed with warm PBS twice and then detached by trypsination. After that, the collected floating cells were washed with cold PBS once and then re‐dispersed in 500 µL of 1× annexin V binding buffer. Subsequently, 1 µL of PI (propidium iodide, dead cells) and 2 µL of Annexin‐V‐FITC (apoptotic cells) were added to the solution. The solution was gently vortexed, reacted at room temperature in the dark for 15 min, were filtered through cell strainers and analyzed by flow cytometry (CytoFLEX S, Beckman Coulter).

### Haemolysis Analysis

Two percent red blood cells were added into different solutions, including deionized water (positive control), PBS (negative control), (COFs@HA (weighing concentration: 10 ppm), COF/Au@HA (Au concentration: 10 ppm), COF/Ir@HA (Ir concentration: 10 ppm), and COF/Au_0.75_/Ir_0.25_@HA (Ir concentration: 10 ppm) nanoparticles). The samples were kept at room temperature in the dark for 30 min. Afterward, hemoglobin levels in the supernatants from various treatments were quantified by measuring the absorbance at 500 nm with a microplate reader. After the assay was completed, the percentage of hemolysis was calculated using the following equation:

(1) $$\begin{array}{ccc} & & \mathrm{Percentage}\;\mathrm{of}\;\mathrm{haemolysis}=\left[\left(\mathrm{absorbancesample}-\mathrm{absorbancecontrol}\right)/\right.\\ & & \left.\left(\mathrm{absorbancemaximal}\;\mathrm{lysis}-\mathrm{absorbancecontrol}\right)\right]\times 100\% \end{array}$$

### DFT Calculations

All DFT calculations were performed using the Vienna ab‐Initio Simulation Package.^[^ ^43^, ^44^, ^45^, ^46^ ^]^ The projector‐augmented‐wave method^[^ ^47^, ^48^ ^]^ was used in conjunction with the generalized gradient‐approximation and Perdew–Burke–Ernzerhof exchange–correlation functional.^[^ ^49^ ^]^ The Kohn–Sham orbitals were expanded in a plane‐wave basis set with a kinetic energy cutoff of 400 eV. The DFT‐D3 dispersion correction was performed to study the effect of van der Waals interaction.^[^ ^50^, ^51^ ^]^ Brillouin zone sampling was conducted using a Gamma point in the calculations. The convergence threshold was set to be 10^−5^ eV for the total electronic energy in the self‐consistent loop. The atomic positions were relaxed using either the conjugate‐gradient (CG) algorithm until the *x*, *y*, *z*‐components of the unconstrained atomic force were smaller than 2 × 10^−2^ eV Å^−1^. The structure of the TAPB‐BTCA‐COF contained the chemical formula of C_99_H_63_N_9_, as shown in Figure 5a, in which the optimized lattice parameters of the COF were *a* = 32.48 Å and *b* = 31.66 Å. The model resembled the previously reported TAPB‐BTCA‐COF optimized structures described in the literature.^[^ ^52^, ^53^ ^]^

### Orthotopic Hepatocellular Carcinoma Animal Model for In Vivo Bioluminescence Imaging of Liver Cancer

The animal experiment of orthotopic cancer was conducted using Hep G2‐Red‐FLuc cells xenograft mouse model. NOD‐SCID mice (6–8 weeks, female) were obtained from the Laboratory Animal Center, College of Medicine at National Cheng Kung University (NCKU). The experimental mice were housed in cages (five mice in each cage) at ≈22–23 °C and 55 ± 10% humidity with a 13 h/11 h light/dark cycle. All animal treatments and surgical procedures were performed in accordance with the guidelines of NCKU Laboratory Animal Center (IACUC No. 113 283). Mice were anesthetized using an intraperitoneal injection of Zoletil 100 (Virbac) and injected 2 × 10^6^ Hep G2‐Red‐FLuc cells in 20 µL of basement membrane matrix (BD) gel mixed into the left side of the liver using surgical methods. Following this procedure, the mice were imaged using bioluminescence. Bioluminescence flux, an indicator of tumour growth, was measured after the administration of d‐luciferin (Biosynth Carbosynth) and recorded using an IVIS (PerkinElmer) system. In accordance with the guidelines set by the Institutional Animal Care and Use Committee of NCKU for survival analysis, survival time was not recorded and the study was terminated upon detecting cancer metastasis based on the ethical guidelines for animal experimentation.

### Haematoxylin and eosin (H&E) Staining, Immunohistochemistry (IHC) Staining of the Ex Vivo Samples from Liver Cancer

The tumor samples or other normal organs (heart, lung, spleen, liver, and kidney) were paraffin‐embedded and sliced into 5 µm‐thick sections. The sections were deparaffinized, rehydrated, and washed with PBS before being stained with hematoxylin solution for 3 min. After rinsing with tap water, the sections were stained with eosin solution for 1 min. Finally, the sections were sequentially immersed in ethanol and xylene before being mounted for evaluation. The prepared sections were then examined under a microscope. For IHC staining, tumor samples were paraffin‐embedded and sectioned into 5 µm‐thick slices. The sections were deparaffinized, rehydrated, and incubated with either a phospho‐histone H2AX antibody (1:400 dilution, GTX636713, GeneTex) or a cleaved caspase‐3 (Asp175) antibody (GTX86952, GeneTex). Staining was performed using an ABC peroxidase standard staining kit (Thermo Fisher Scientific), which included biotinylated affinity‐purified goat anti‐rabbit IgG (1:1000 dilution, 32 054, Thermo Fisher Scientific) and a DAB peroxidase (HRP) substrate kit (Vector Laboratories), following the manufacturer's instructions.

### Blood Biochemical Analysis

Blood samples were collected from the mice via cardiac puncture, and heparin sodium was added immediately to prevent clotting. The samples were centrifuged at 100 × g for 10 min to separate the serum. The serum was then analyzed for blood biochemistry markers, including alkaline phosphatase (ALP), total bilirubin (T‐BIL), blood urea nitrogen (BUN), creatinine (CREA), and uric acid (UA), using a FUJI DRI‐CHEM 4000i analyzer (FUJIFILM).

### In Vivo Biodistribution Analysis

NOD‐SCID mice (6–8 weeks old) were obtained from the Laboratory Animal Center, National Cheng Kung University, and established the Hep G2 xenograft liver tumor. The COF/Au_0.75_/Ir_0.25_@HA NPs (100 µg mouse^−1^, 50 µL) were intravenously administered to tumor‐bearing mice (*n* = 3 per group). Major organs (heart, liver, spleen, lungs, and kidneys) and tumor samples were collected, weighed, and analyzed for Ir content using inductively coupled plasma atomic emission spectroscopy (ICP‐AES).

### Statistical Analysis

Statistical analysis was conducted using GraphPad Prism 8 (GraphPad Software) and Microsoft Excel 2016. Data were analyzed using Student's *t*‐test or one‐way analysis of variance (ANOVA). A P value of less than 0.05 was considered statistically significant.

## Conflict of Interest

The authors declare no conflict of interest.

## Supporting information



Supporting Information

## Data Availability

The data that support the findings of this study are available in the supplementary material of this article.
