# Supplementary material for: Enhanced Catalytic Cycle of Glucose Oxidation and Reactive Species with ROS and RHS Generation Mediated by Galvanic Engineering of Dual Atomic Sites on Covalent Organic Frameworks Demonstrating Synergistic Bimetal Tumor Treatment
Source: Adv Sci (Weinh). 2025 May 28;12(29):e00515. doi: 10.1002/advs.202500515 (PMC12362782; doi:10.1002/advs.202500515)
Supplement: Supplementary file 1 — Supporting Information [file ADVS-12-e00515-s001.docx]

Supporting Information

Enhanced Catalytic Cycle of Glucose Oxidation and Reactive Species with ROS and RHS Generation Mediated by Galvanic Engineering of Dual Atomic Sites on Covalent Organic Frameworks Demonstrating Synergistic Bimetal Tumor Treatment

Wei-Chung Pan, Cheng-Hung Luo, Wen-Ling Lin, Liu-Chun Wang, Divinah Manoharan, Po-Ya Chang, Hwo-Shuenn Sheu*, Chen-Hao Yeh*,Chia-Jui Yen*, and Chen-Sheng Yeh*

**Table S1.** Curve-fit parameter for Au *L_3_*-edge EXAFS of sample COF/Au

|  | **Path^b^** | **CN** | **R (Å)** | **σ^2^ (Å^2^)** |
| --- | --- | --- | --- | --- |
| Au foil | Au-Au1 | 12^c^ | 2.86(1) | 0.008(6) |
|  | Au-Au2 | 6^c^ | 4.08(3) | 0.012(4) |
|  | Au-Au1-Au1 | 48^c^ | 4.24(8) | 0.008(14) |
| COF/Au | Au-Au1 | 9.24 | 2.79(1) | 0.012(1) |

^a^ The S0^2^ factor (0.8286) of sample COF/Au was applied using a reference Au foil. ΔE_0_ was refined as a global fit parameter, returning a value of 2.3 ± 0.7 eV. The data range covered 3 ≤ *k* ≤ 11 Å^-1^ and 1.5 ≤ *R* ≤ 3 Å. There were 4 variable parameter out of a total of 7 independent points. The R factor for this fit was 0.80%. ^b^ The distances for Au-Au1 was from the CIF of Au(ICSD163723). ^c^ The coordination numbers were constrained as follow: N(Au-Au1)=12, N(Au-Au2)=6, and N(Au-Au1-Au1)=48 to determine the relative S0^2^ applied to the sample COF/Au.

**Table S2.** Curve-fit Parametera for Au *L_3_*-edge EXAFS for COF/Au/Ir.

|  | **Path^b^** | **CN** | **R (Å)** | **σ^2^ (Å^2^)** |
| --- | --- | --- | --- | --- |
| Au foil | Au-Au1 | 12^c^ | 2.86(1) | 0.008(6) |
|  | Au-Au2 | 6^c^ | 4.08(3) | 0.012(4) |
|  | Au-Au1-Au1 | 48^c^ | 4.24(8) | 0.008(14) |
| COF/Au/Ir | Au-Cl1 | 2.05 | 2.26(1) | 0.002(1) |

^a^ The S0^2^ factor (0.7532) of sample COF/Au/Ir was applied using a reference Au foil. ΔE_0_ was refined as a global fit parameter, returning a value of 4.4 ± 0.9 eV. The data range covered 3 ≤ *k* ≤ 12 Å^-1^ and 1 ≤ *R* ≤ 3 Å. There were 4 variable parameter out of a total of 11 independent points. The R factor for this fit was 1.45%. ^b^ The distances for Au-Cl1 was from the CIF of AuCl_3_ (ICSD5467). ^c^ The coordination numbers were constrained as follow: N(Au-Au1)=12, N(Au-Au2)=6, and N(Au-Au1-Au1)=48 to determine the relative S0^2^ applied to the sample COF/Au/Ir.


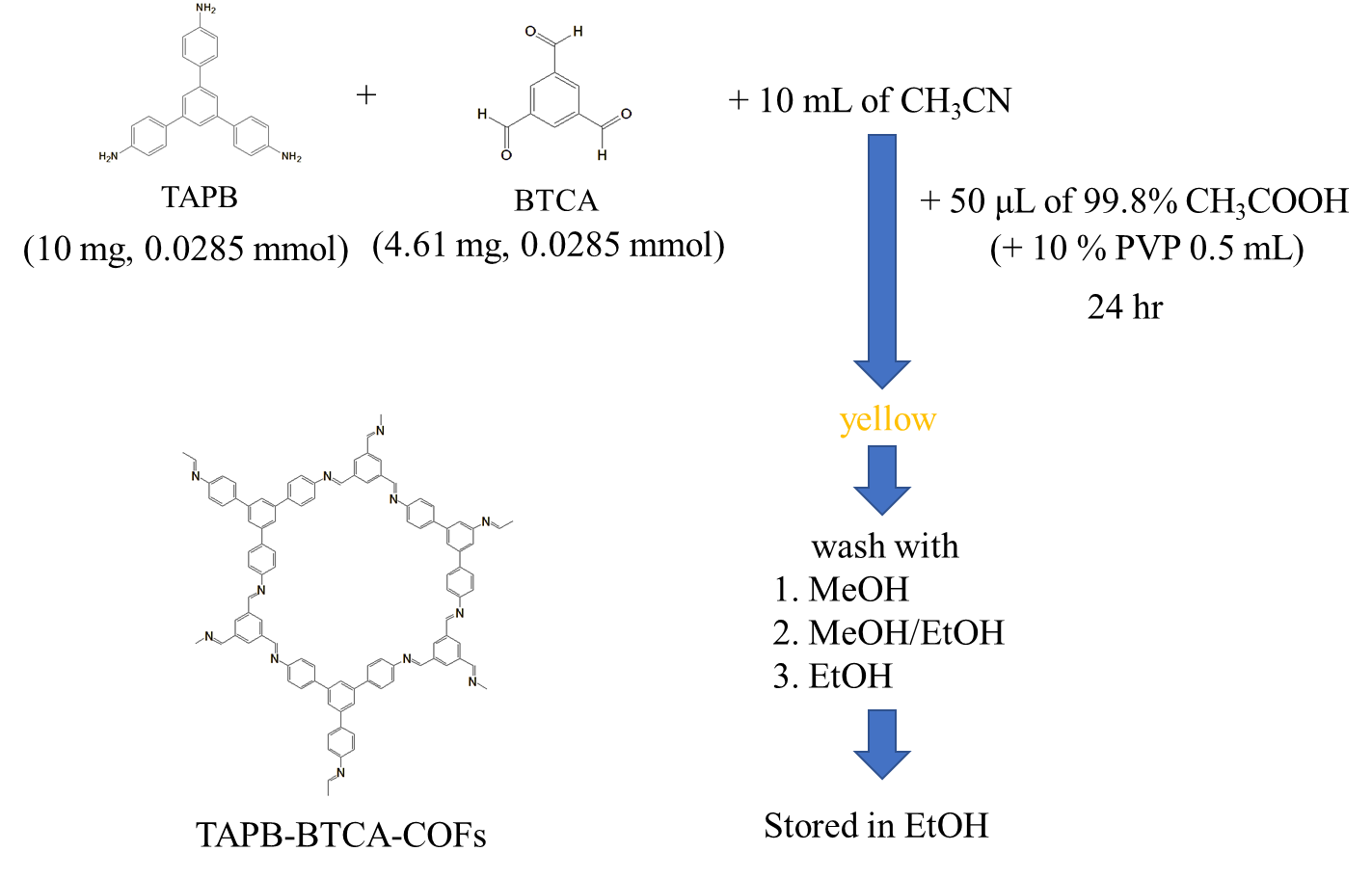


**Figure S1.** Synthesis processes of COF NPs.


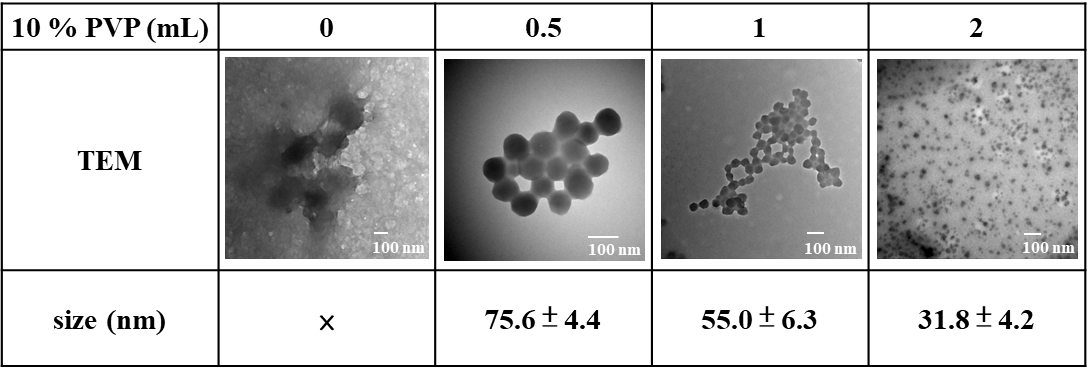


**Figure S2.** TEM images of different sizes of COF NPs through the adjustment of PVP.


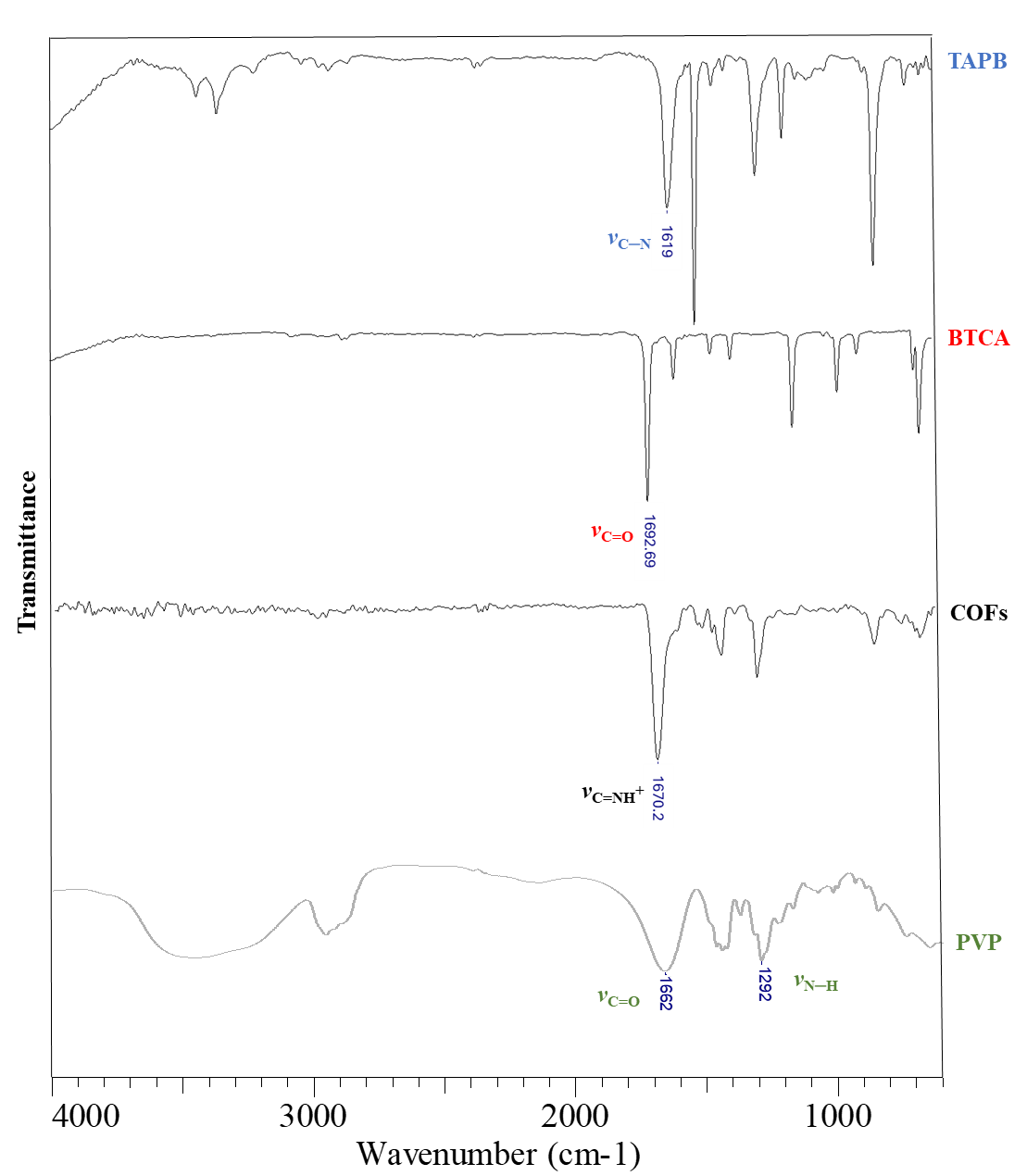


**Figure S3.** FTIR spectra of TAPB, BTCA, COF NPs and PVP.


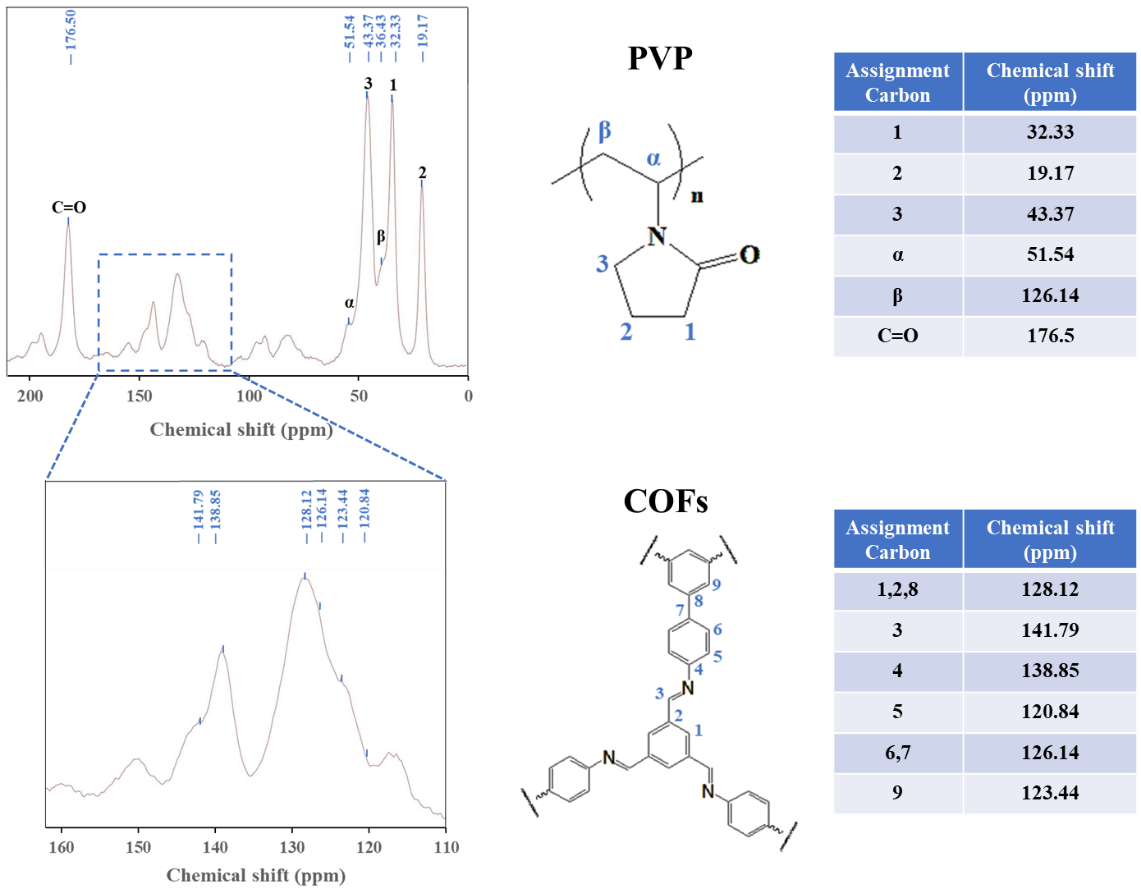


**Figure S4.** Solid-state ^13^C-NMR spectrum of COF NPs. The characteristic signals showed the presence of PVP and COFs.


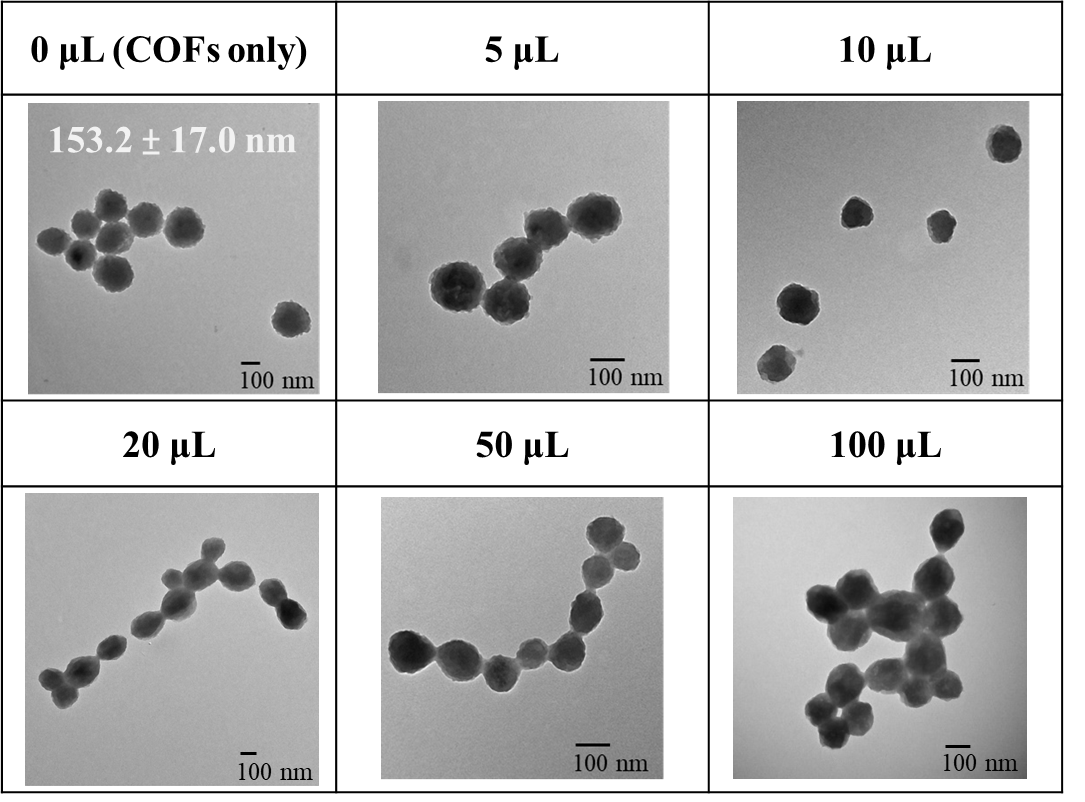


**Figure S5.** TEM images of COF/Ir NPs synthesized from different volumes of 100 mM IrCl_3(aq)_.


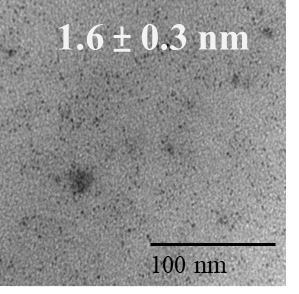


**Figure S6.** TEM image showing pure Ir NPs in the absence of COF NPs for comparison.


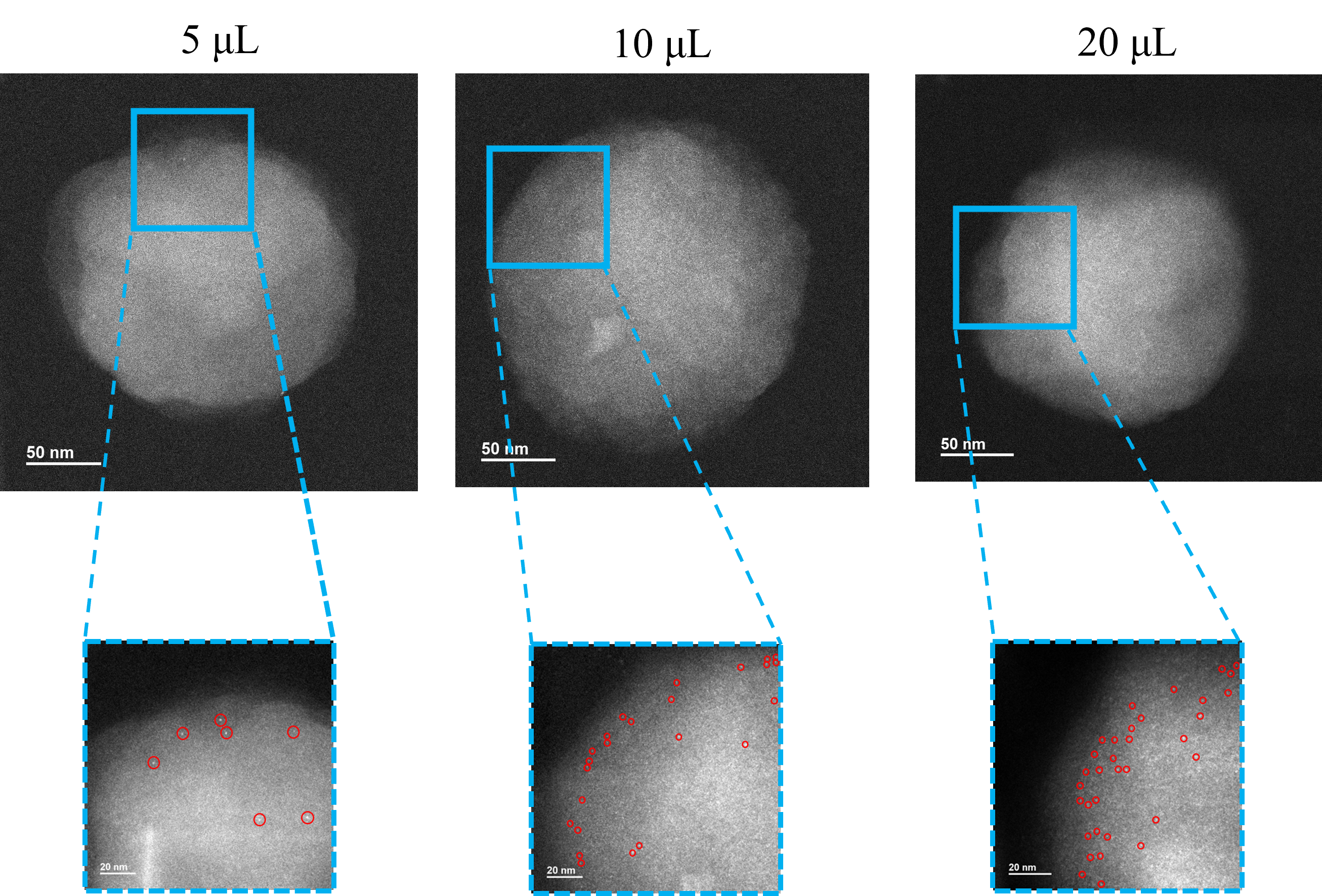


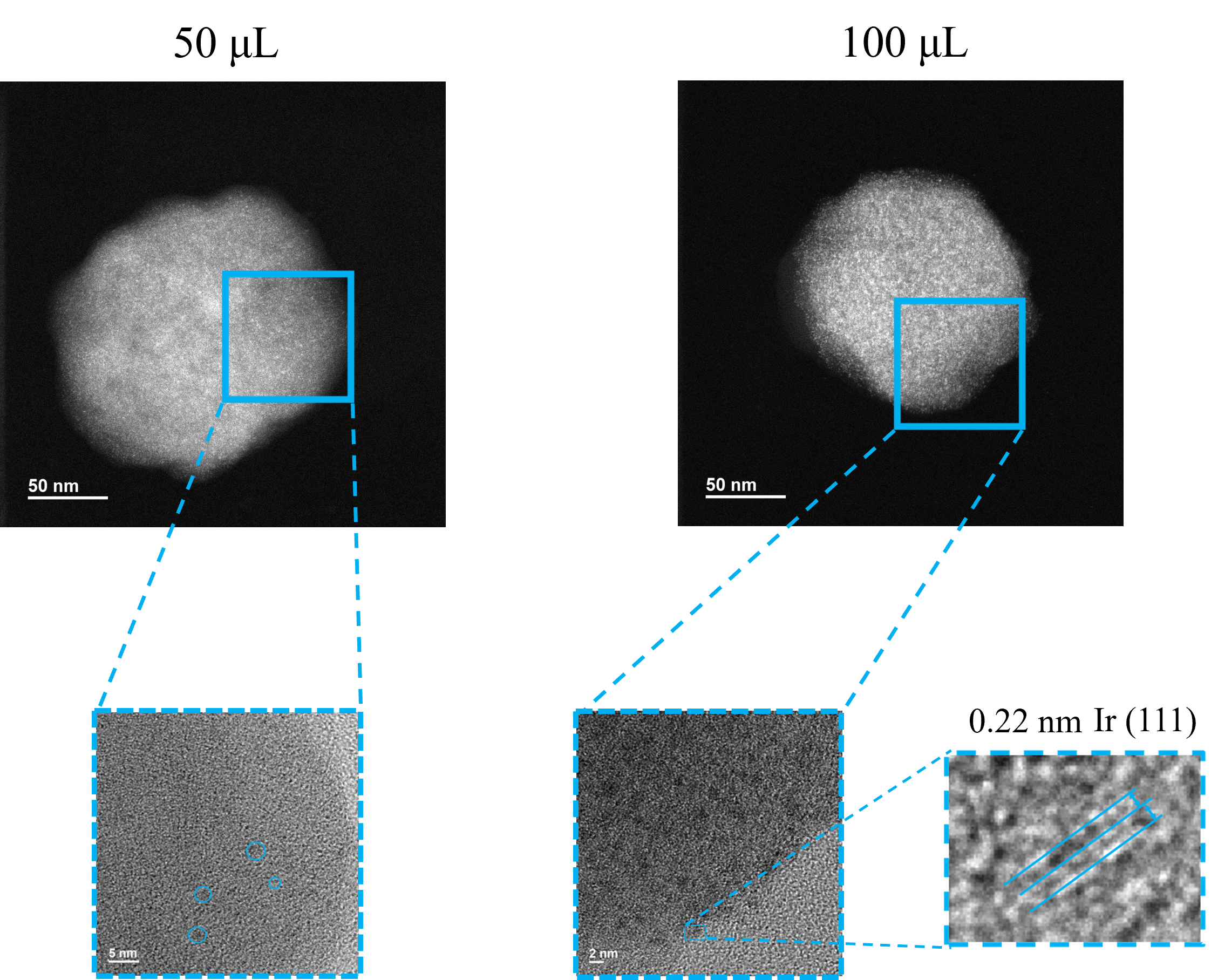


**Figure S7.** AC HAADF-STEM images and high-resolution TEM images of COF/Ir NPs synthesized from different volumes of 100 mM IrCl_3(aq)_. Ir atoms are marked with red circles.


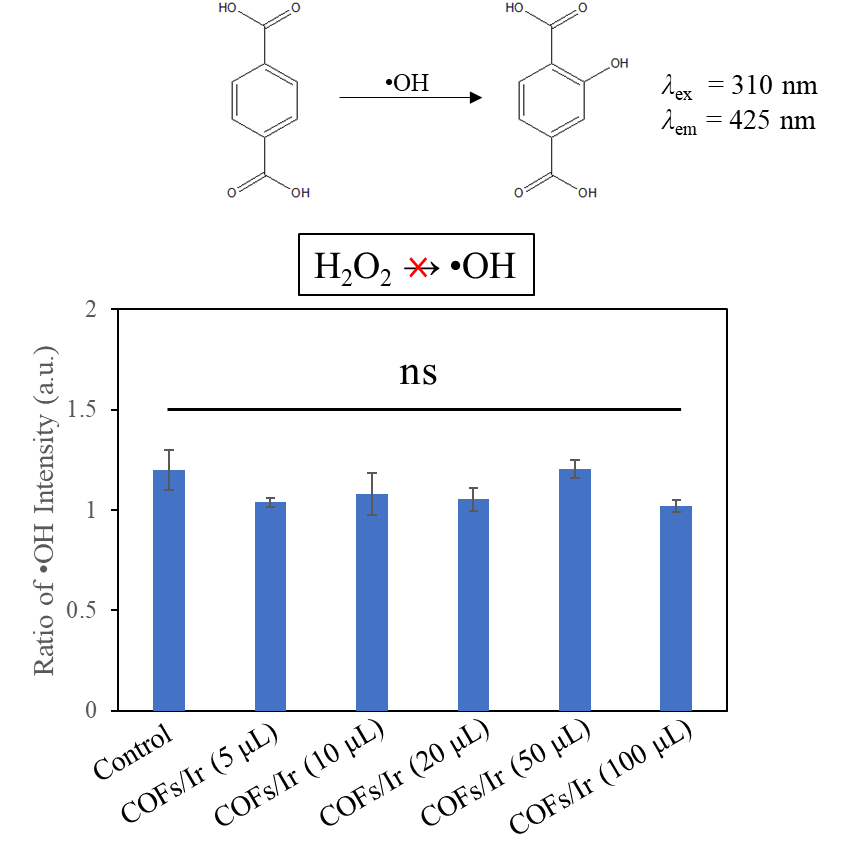


**Figure S8.** Detection of hydroxyl radical (•OH) generation using terephthalic acid showing no •OH production of COF/Ir (different volumes of Ir salt).


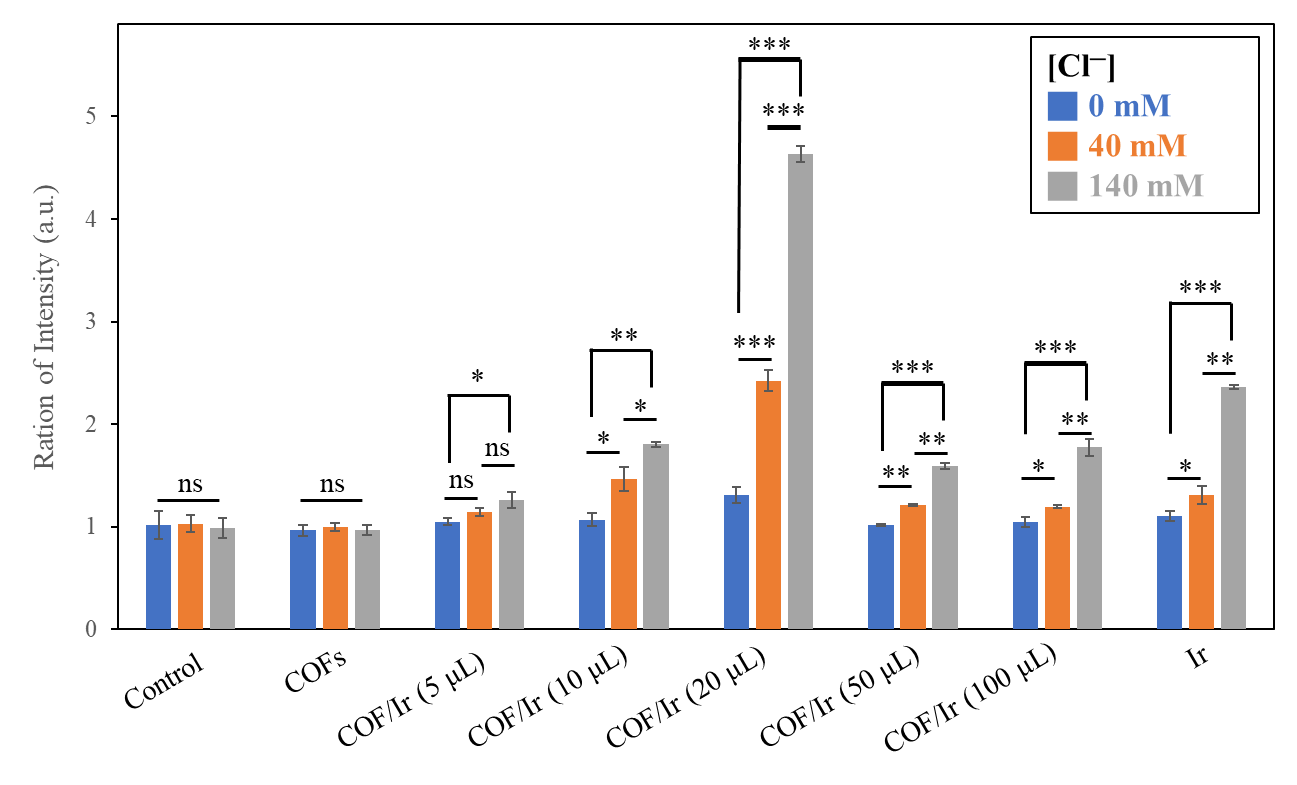


**Figure S9.** HOCl generation of COFs, Ir and COF/Ir NPs under different chloride ion concentrations.


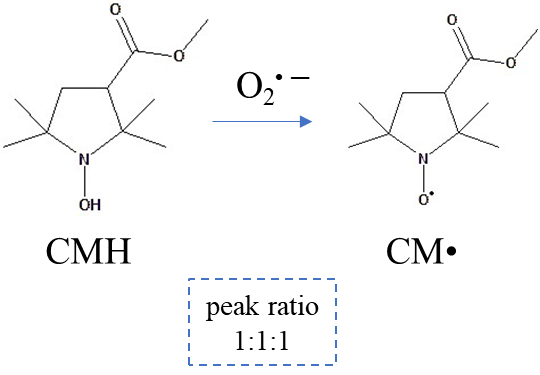


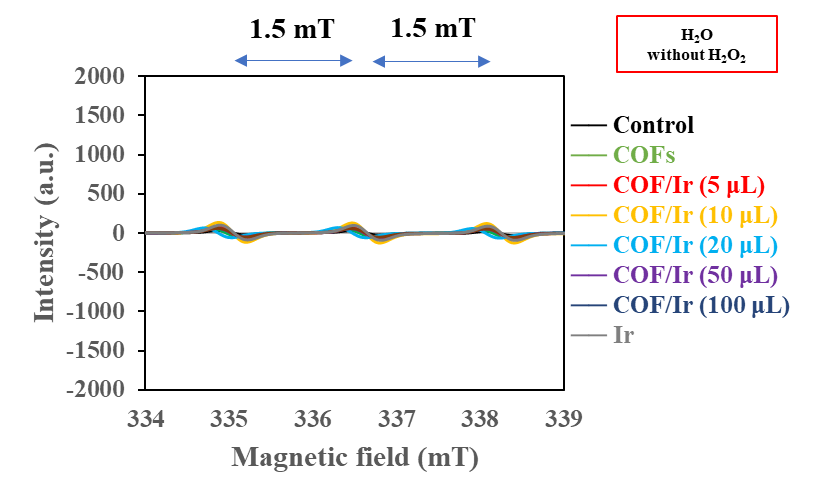

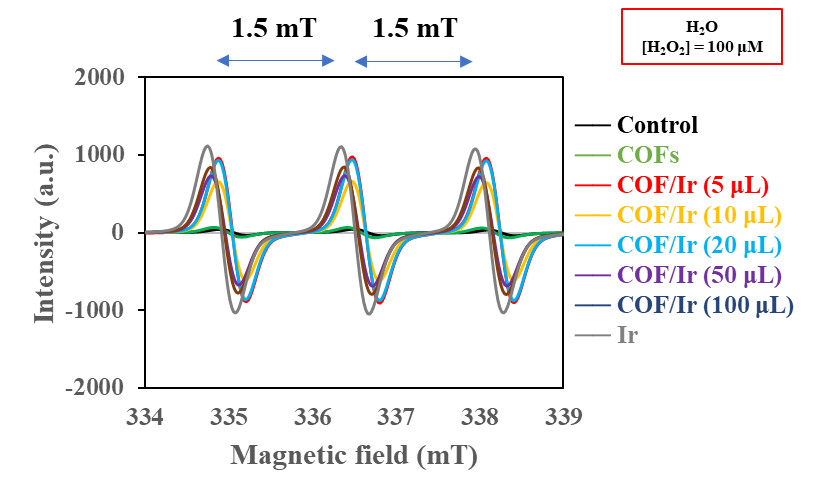


**Figure S10.** Determination of superoxide anion (O_2_ ^• ─^) generation using 1-hydroxy-3-methoxycarbonyl-2,2,5,5-tetramethylpyrrolidine (CMH) reveals the COF/Ir and Ir NPs do not generate O_2_ ^• ─^ without addition of H_2_O_2_.


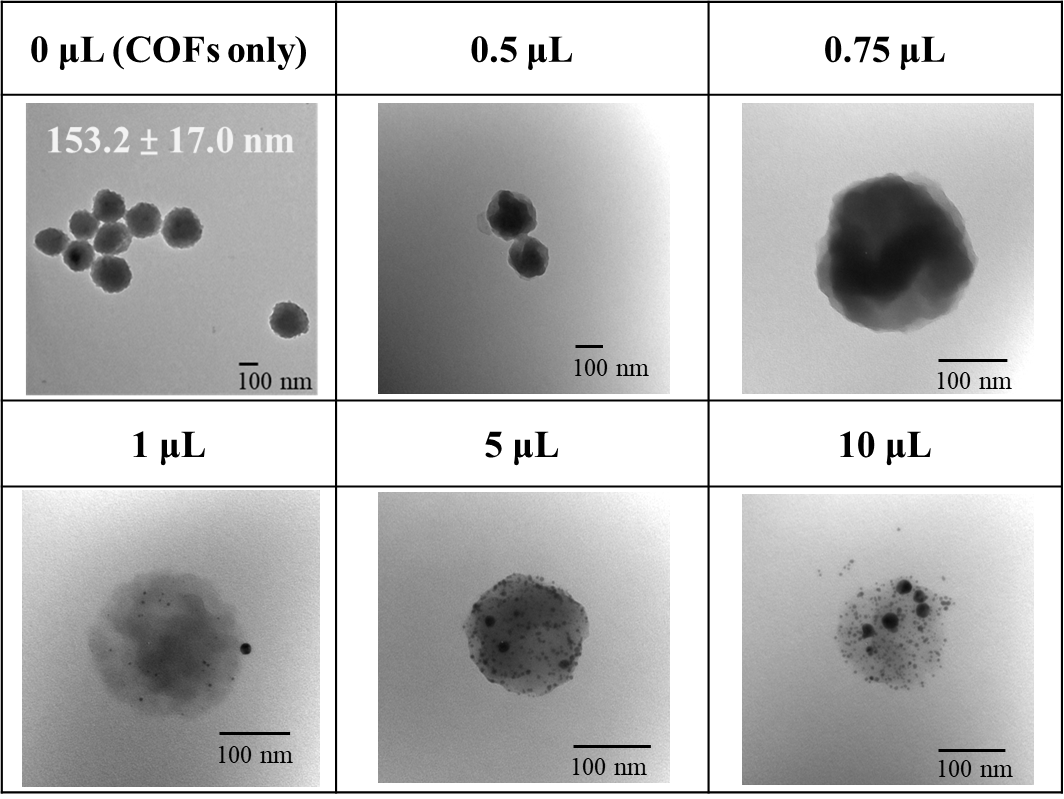


**Figure S11.** TEM images of COF/Au NPs synthesized from different volumes of 50 mM HAuCl_4(aq)_.


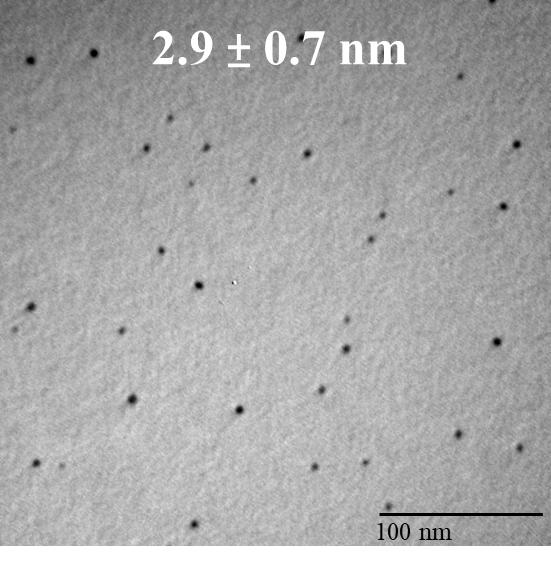


**Figure S12.** TEM images of Au NPs without COFs NPs for comparison.


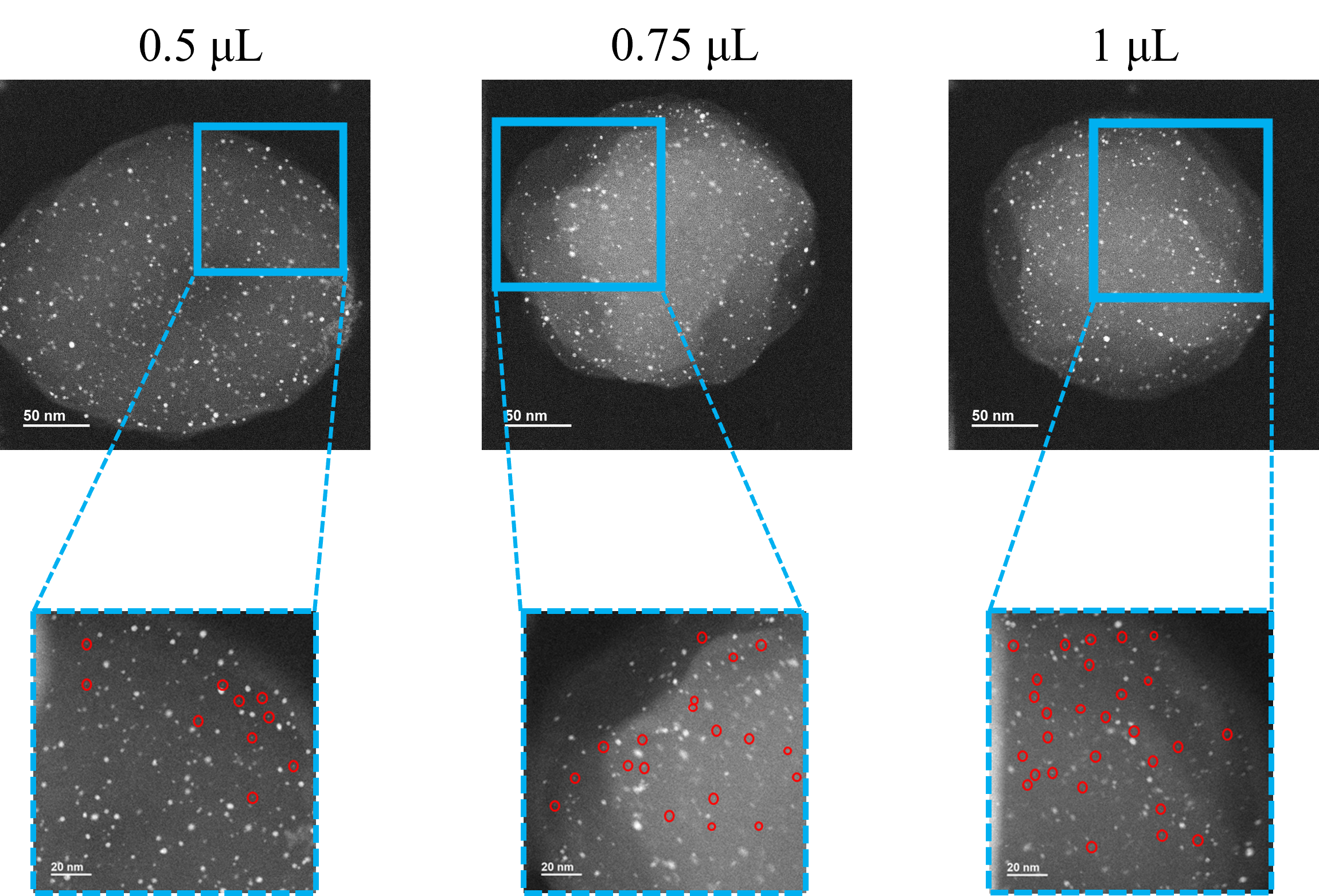


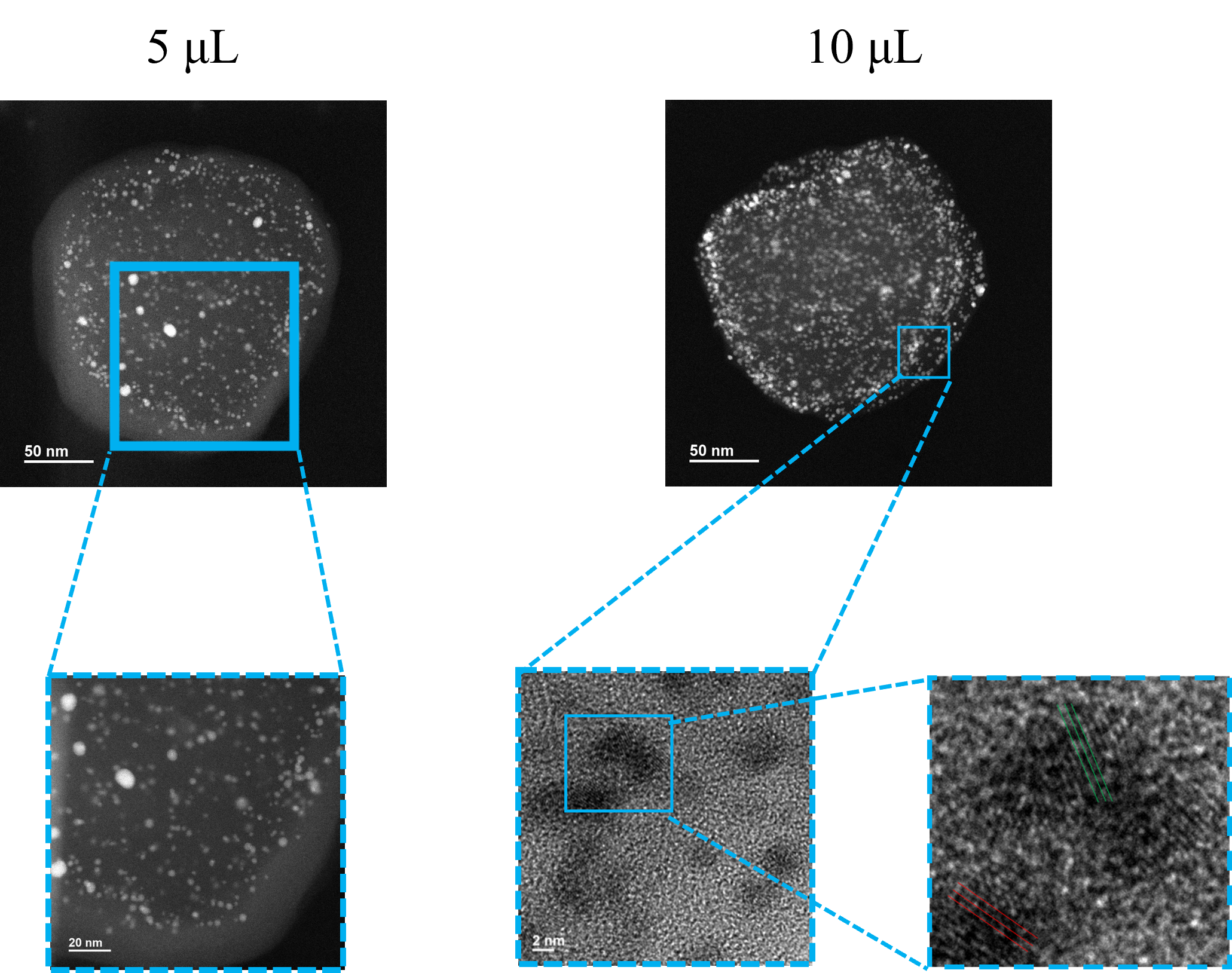


**Figure S13.** AC HAADF-STEM images and high-resolution TEM images of COF/Au NPs synthesized from different volumes of 50 mM HAuCl_4(aq)_. Au atoms are marked with red circles.


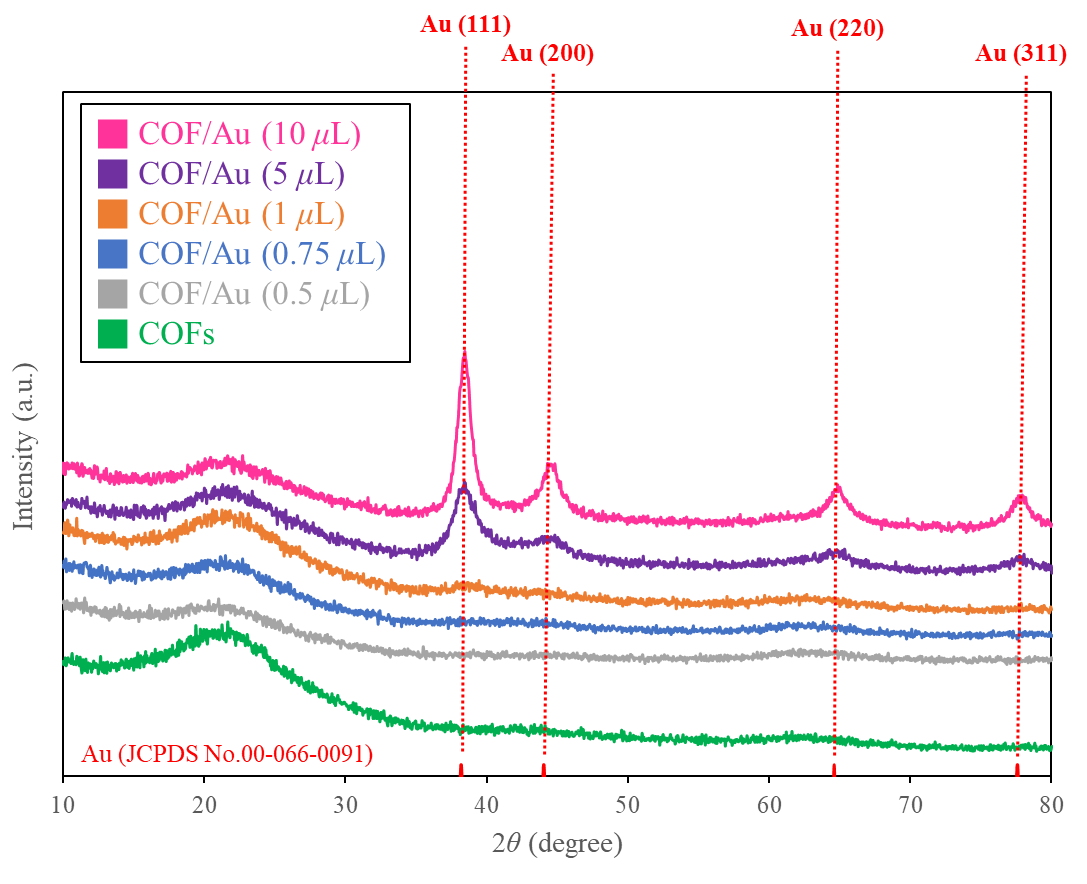


**Figure S14.** XRD patterns of COFs and COF/Au NPs synthesized from different volumes of 50 mM HAuCl_4(aq)_.


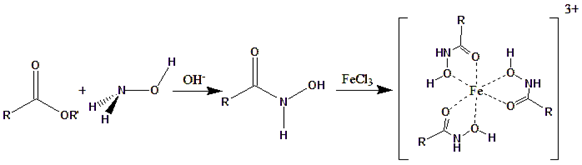


**Figure S15.** The mechanism of forming hydroxamate-Fe³⁺.


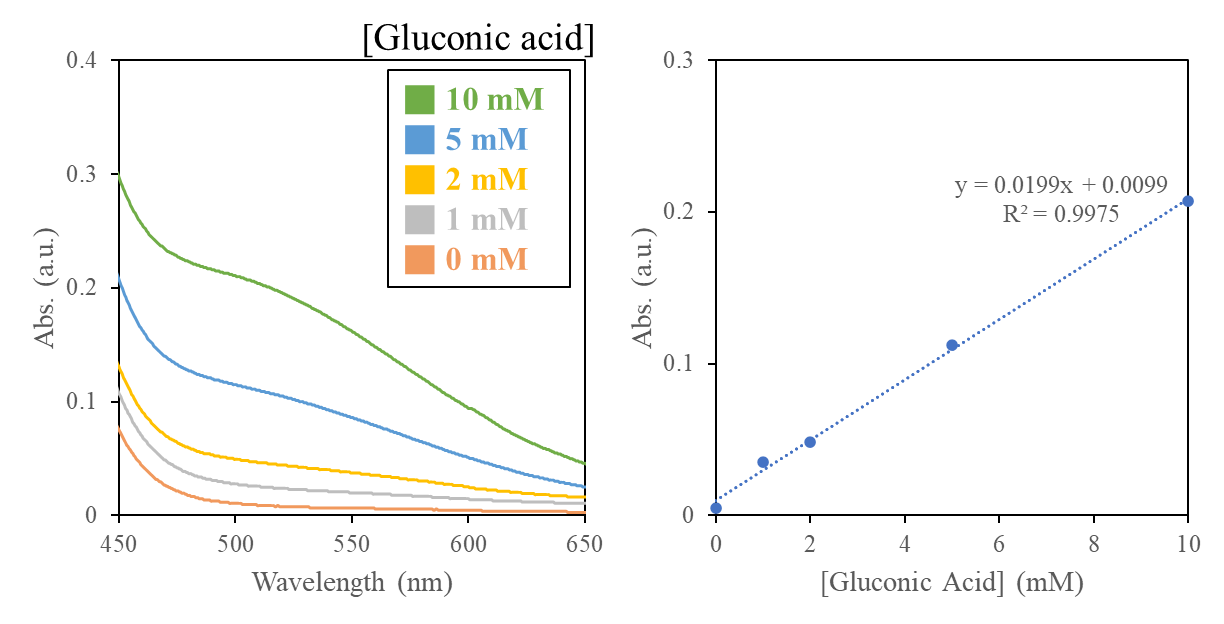


**Figure S16.** UV–Vis profiles of different concentrations of gluconic acid and calibration curve. The calibration curve was obtained from 505 nm absorbance of hydroxamate-Fe³⁺ vs corresponding concentration of gluconic acid.


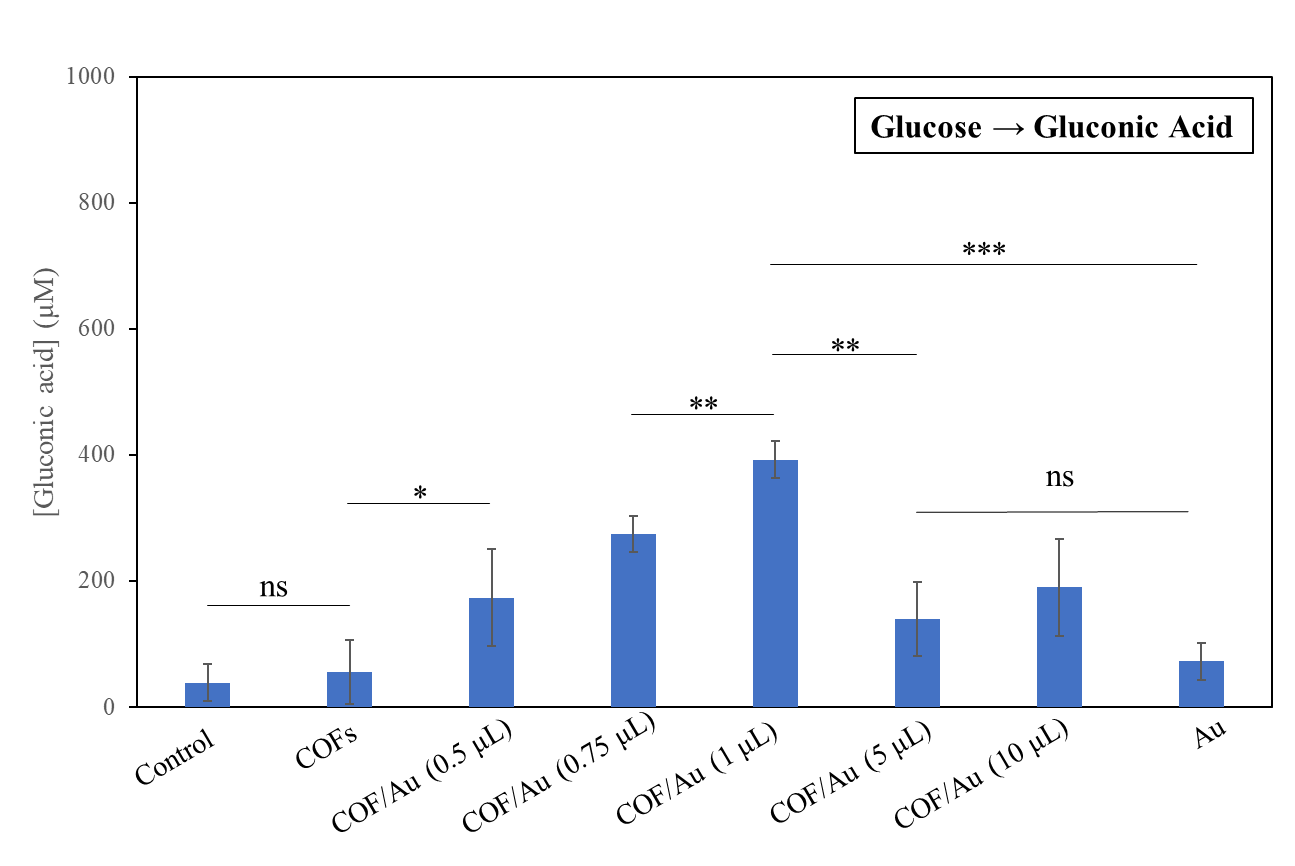


**Figure S17.** Gluconic acid generation of COFs, COF/Au (different volumes of Au salt) and Au NPs.


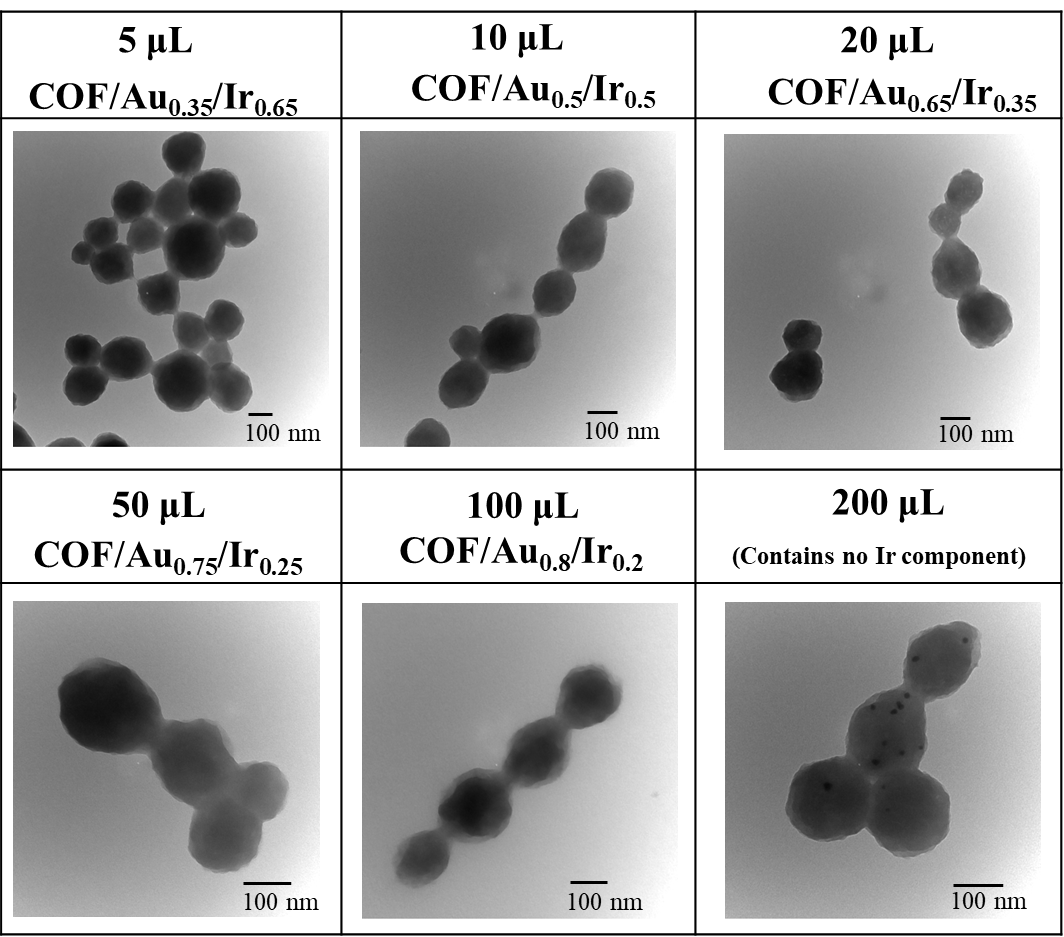


**Figure S18.** TEM images of different ratios of Au to Ir on COF/Au_x_/Ir_1-x_ by adjusting the amounts of 50 mM HAuCl₄.


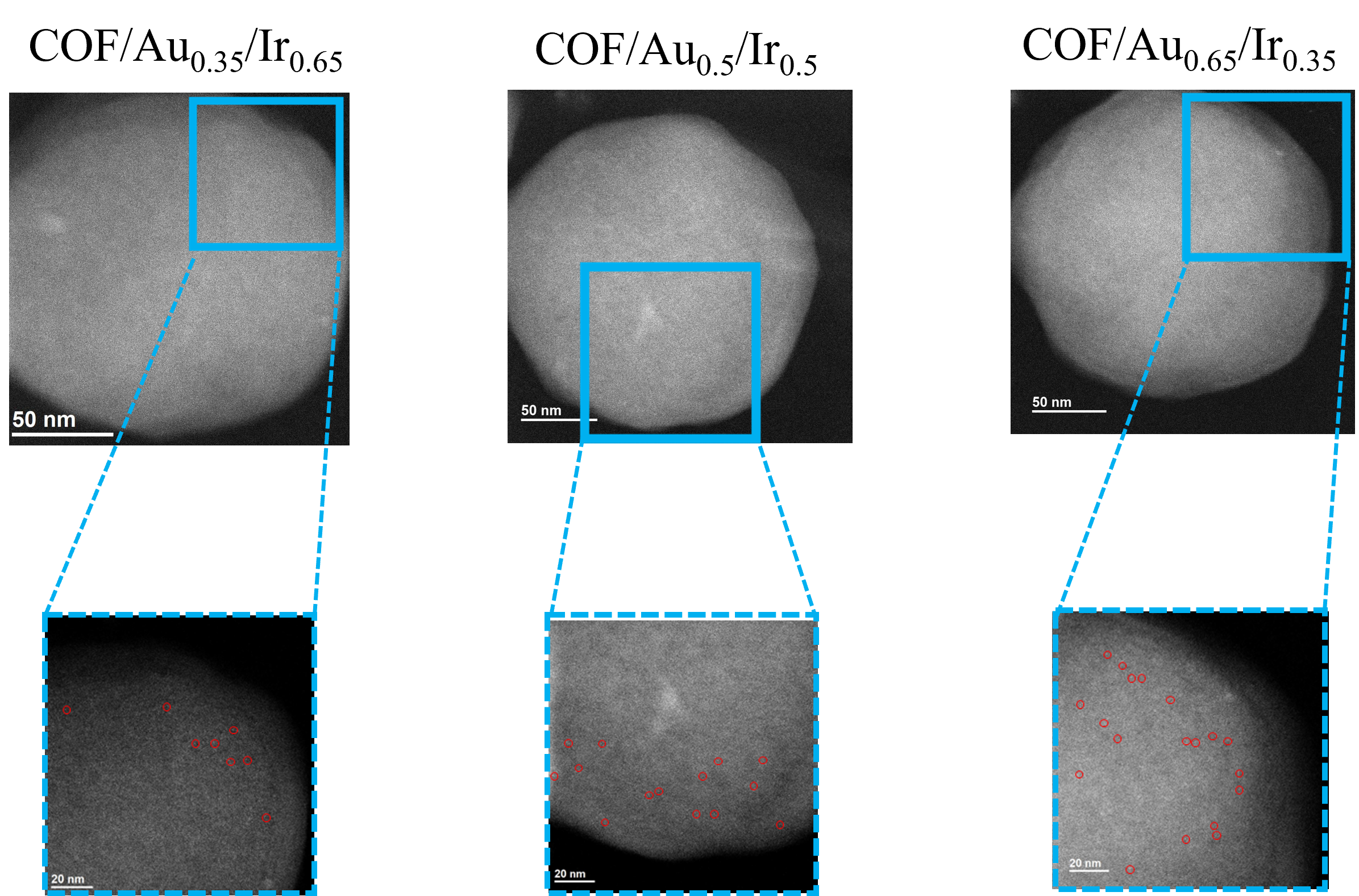


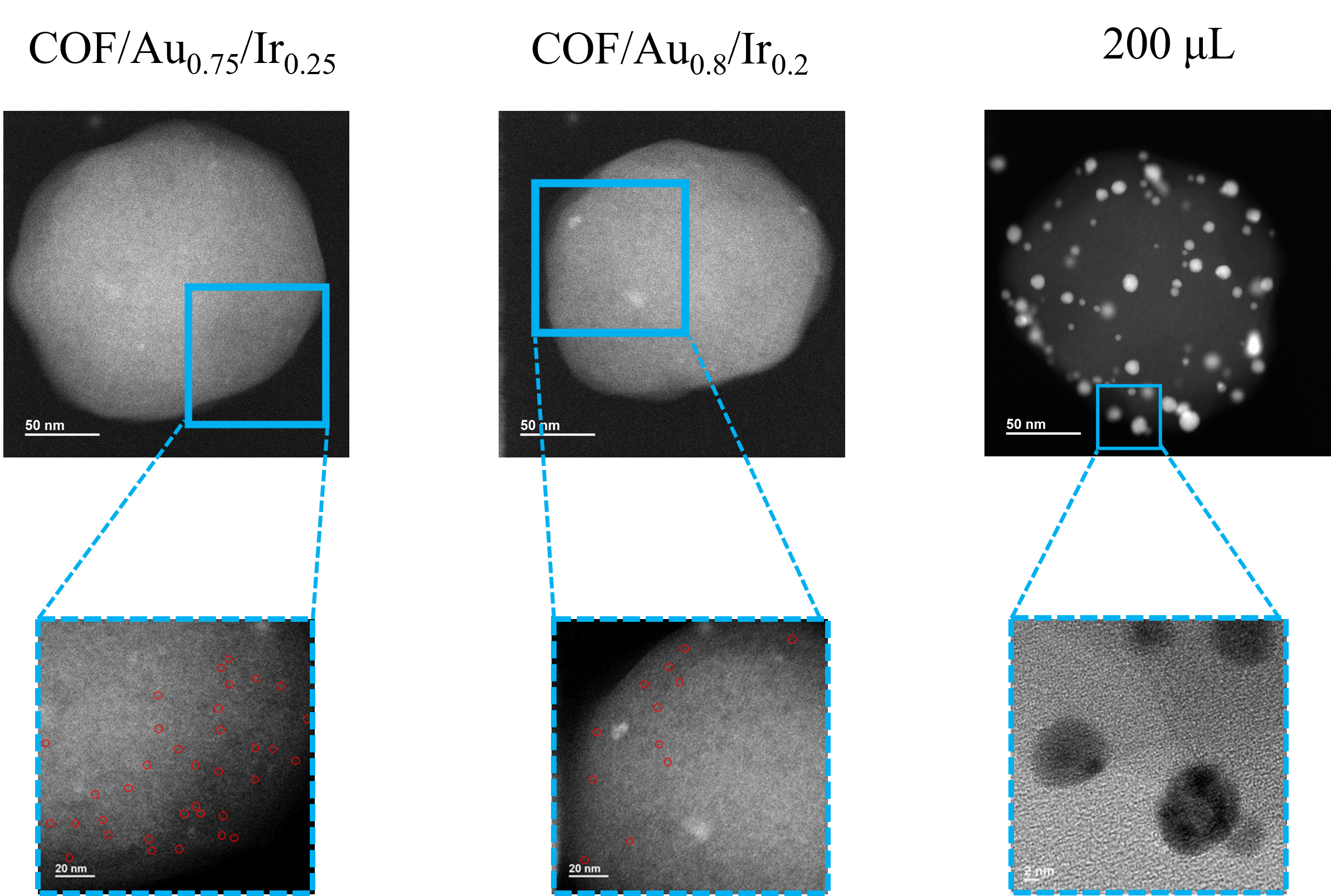


**Figure S19.** AC HAADF-STEM images and high-resolution TEM images of different ratios of Au to Ir on COF/Au_x_/Ir_1-x_ by adjusting the amounts of 50 mM HAuCl₄. Only Au NPs were observed when HAuCl₄ reached 200 μL. Single atoms are marked red circles.


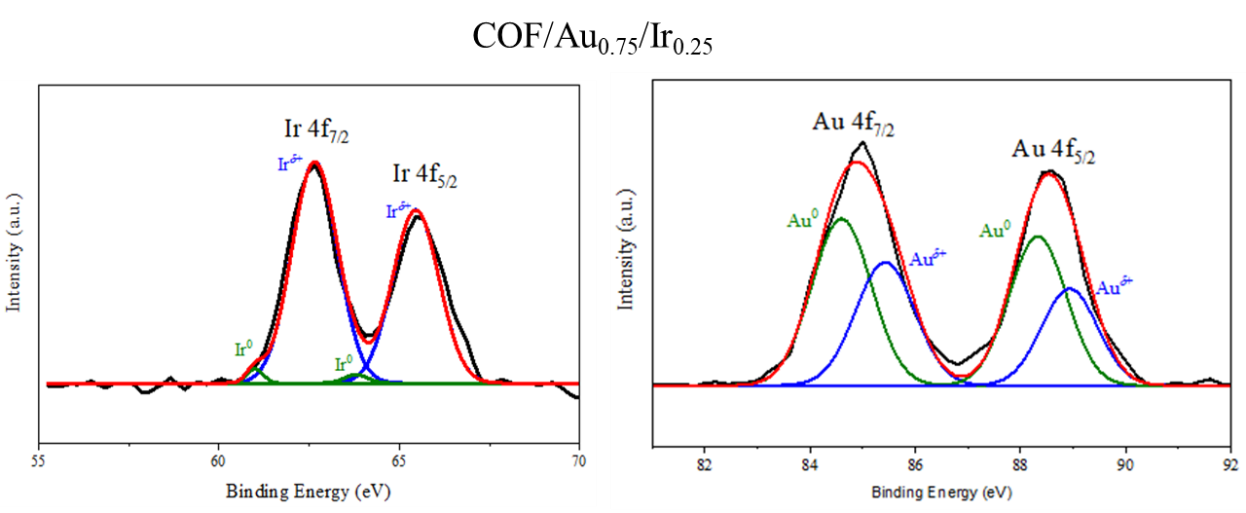


**Figure S20.** XPS analysis of Ir and Au elements on COF/Au_0.75_/Ir_0.25_ NPs.

| 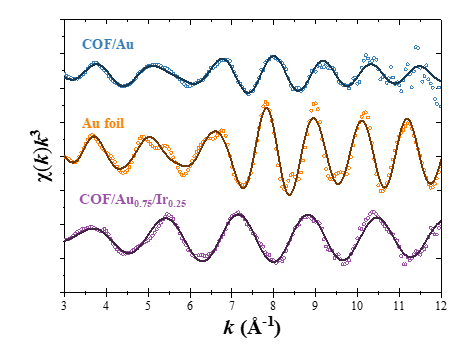 |
| --- |
| **Figure S21.** Au L₃-edge k³-weighted EXAFS spectra of Au foil, COF/Au, and COF/Au_0.75_/Ir_0.25_. The hollow circles represent the experimental data, while the solid line corresponds to the fitting curve. |

|  |
| --- |
|  |


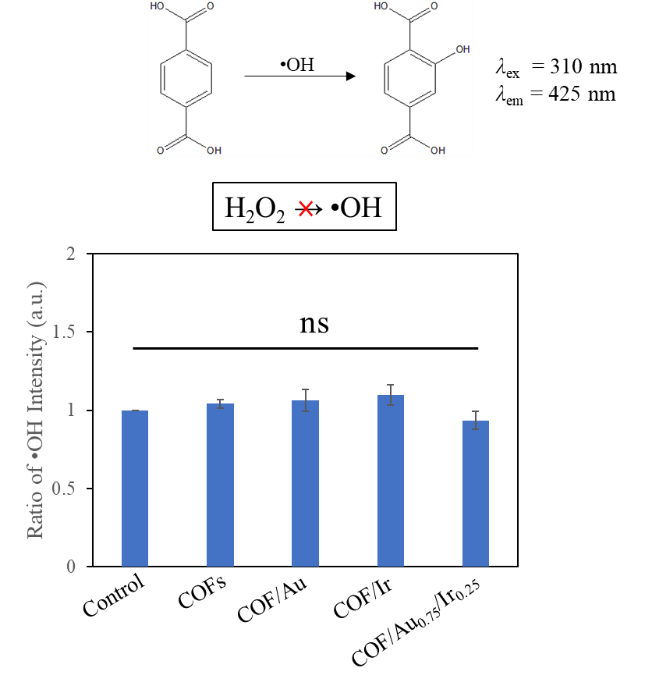


**Figure S22.** Hydroxyl radical (•OH) generation of COFs, COF/Au, COF/Ir, COF/Au_0.75_/Ir_0.25_ NPs showing no •OH production.


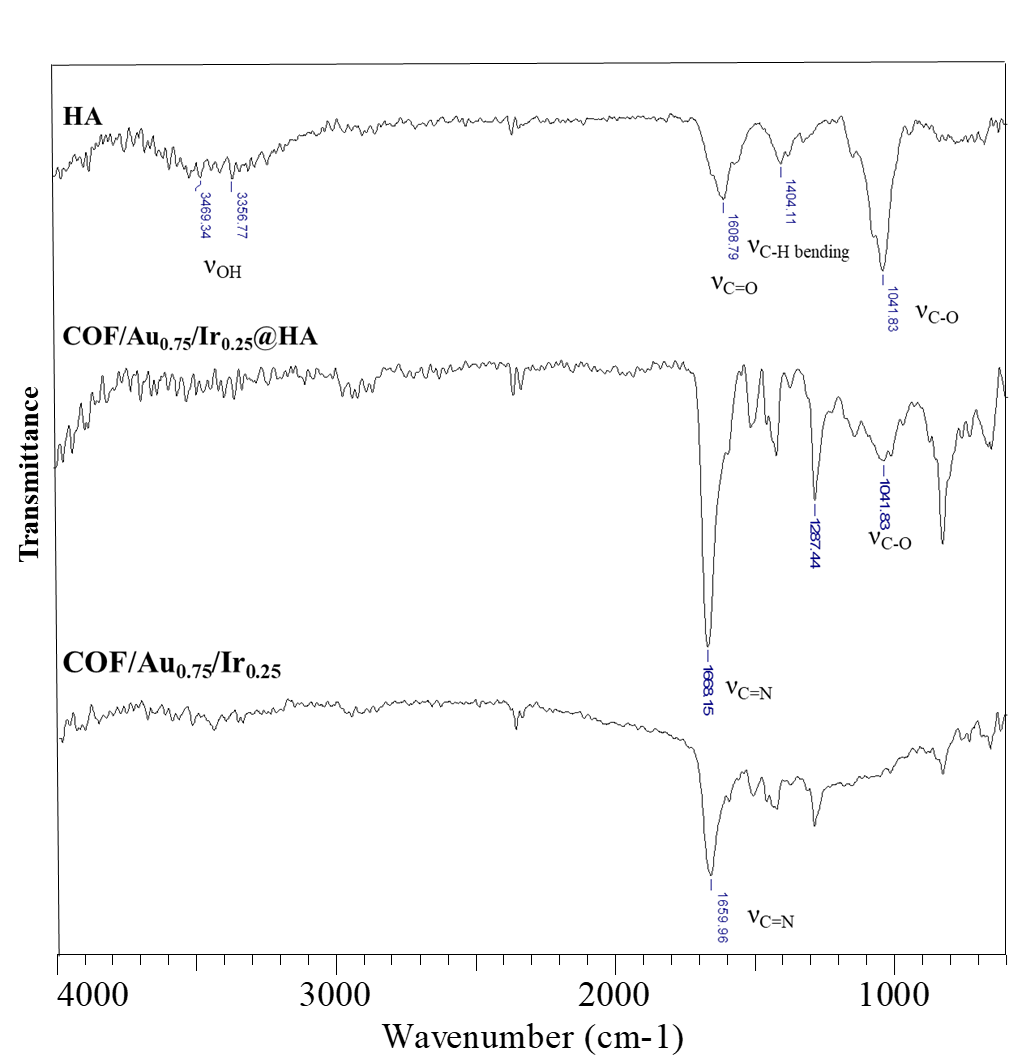


**Figure S23.** FTIR spectra of hyaluronic acid (HA), COF/Au_0.75_/Ir_0.25_ and COF/Au_0.75_/Ir_0.25_@HA NPs.


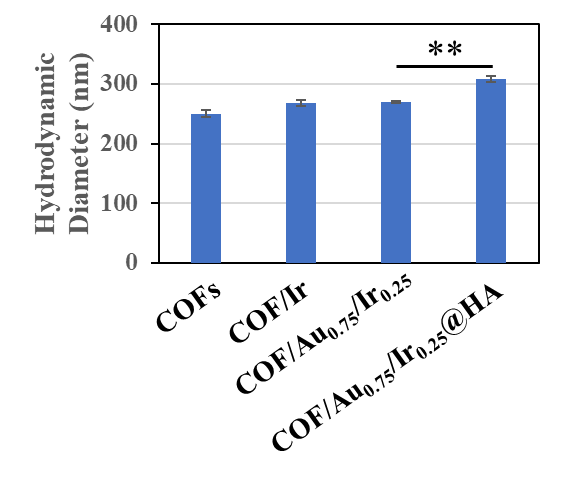


**Figure S24.** The hydrodynamic diameters of COFs, COF/Ir, COF/Au_0.75_/Ir_0.25_ and COF/Au_0.75_/Ir_0.25_@HA NPs.


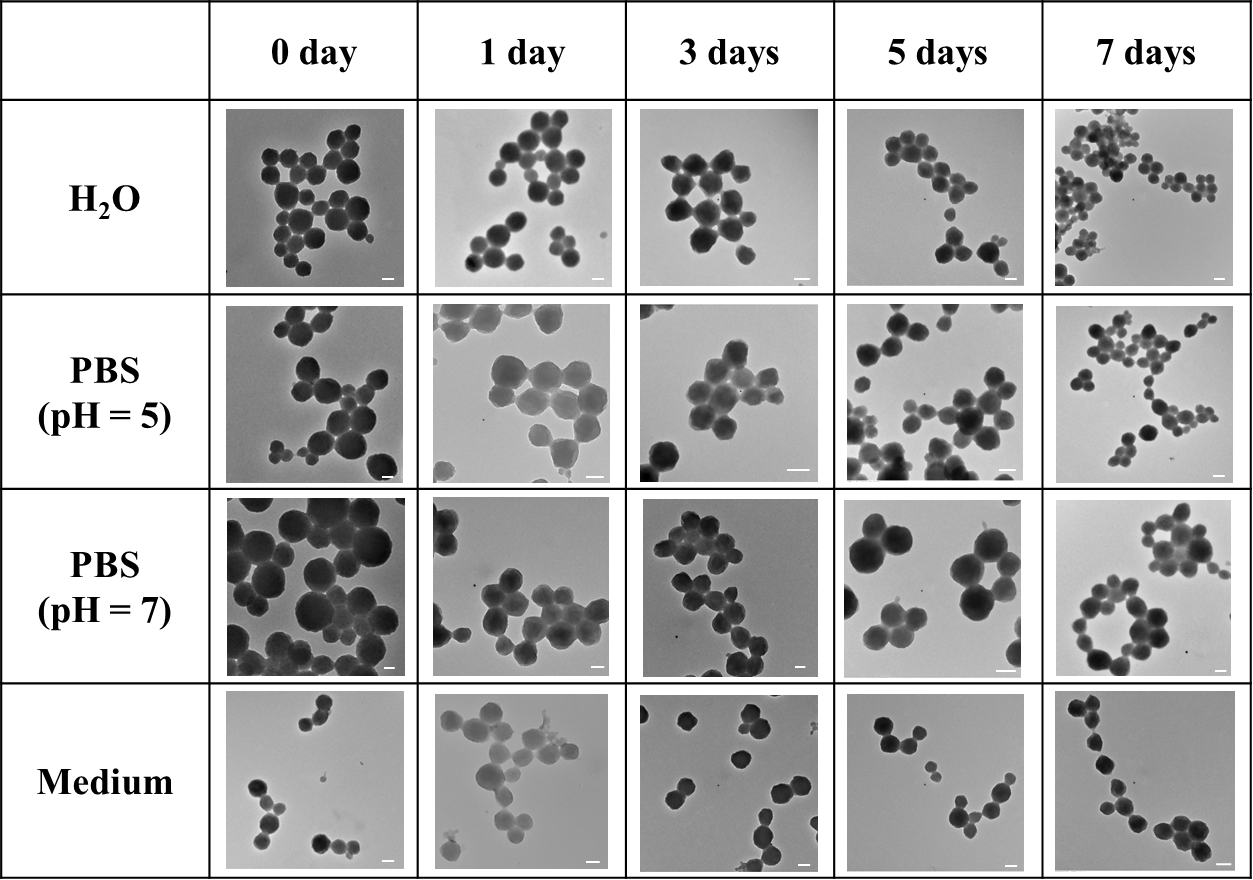


**Figure S25.** Stability of COF/Au_0.75_/Ir_0.25_@HA under different physiological conditions in H_2_O, PBS (pH: 5 and 7) and 10% fetal bovine serum (FBS) medium (scale bar: 100 nm).


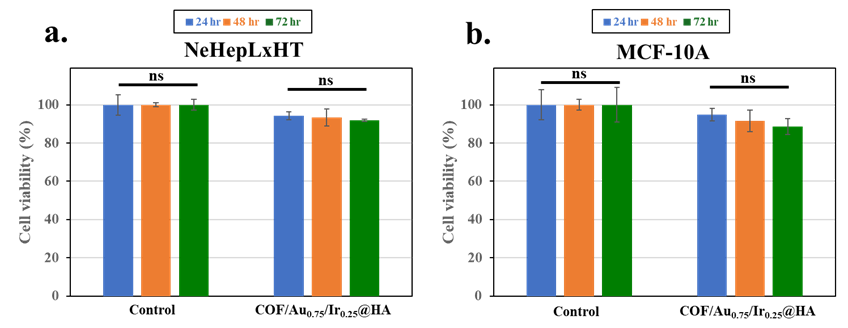


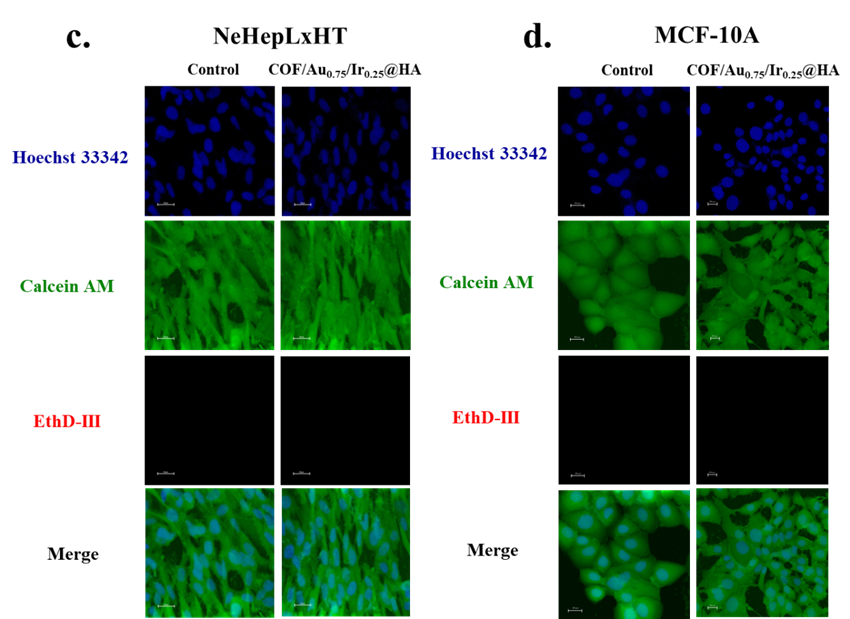


**Figure S26.** The *in vitro* examination of normal NeHepLxHT and MCF-10A (M10) cells incubated with COF/Au_0.75_/Ir_0.25_@HA NPs ([Ir] fixed at 200 ppm). The MTT assay of a) NeHepLxHT and b) M10 cells co-cultured with NPs showing nontoxicity within 3 days. The live and dead staining of c) NeHepLxHT and d) M10 cells (incubation time: 3 days).


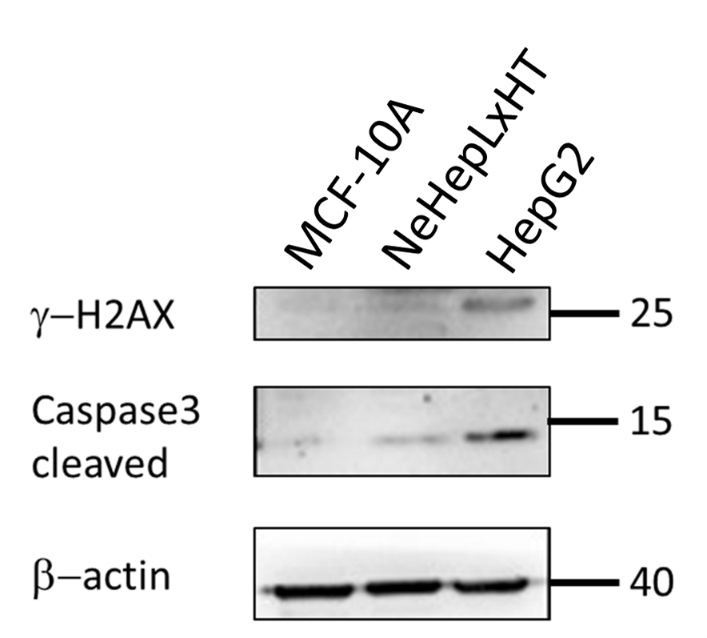


**Figure S27.** The expression of apoptosis signal (γ-H2AX and Caspase3 cleavage form) in MCF-10A, NeHepLxHT, and HepG2 cell lines analyzed by western blotting using anti- γ-H2AX and Caspase3 cleavage form antibody.


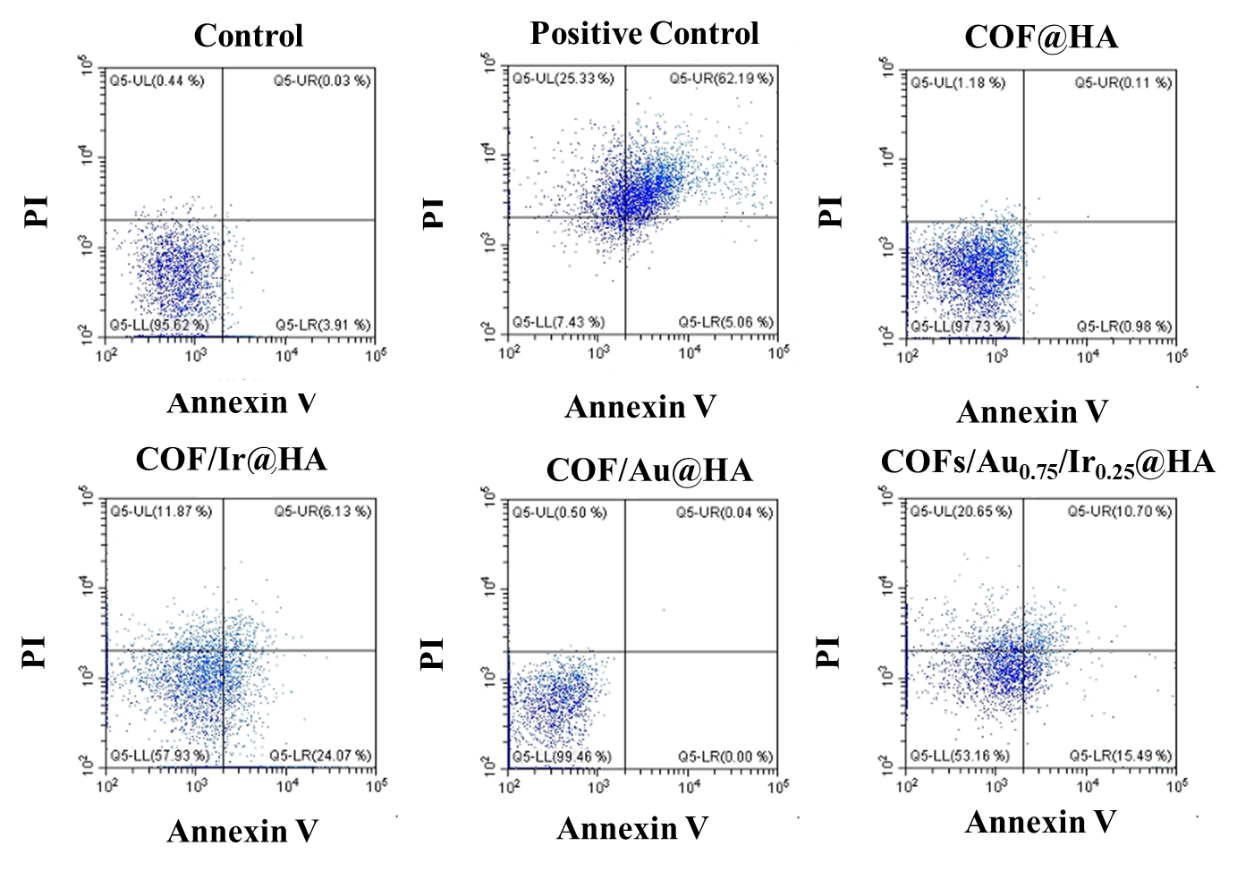


**Figure S28.** Flow cytometry analysis of COF@HA, COF/Ir@HA, COF/Au@HA, and COF/Au₀.₇₅/Ir₀.₂₅@HA NPs after 72 h of incubation with cancer cells, with thapsigargin used as the positive control.


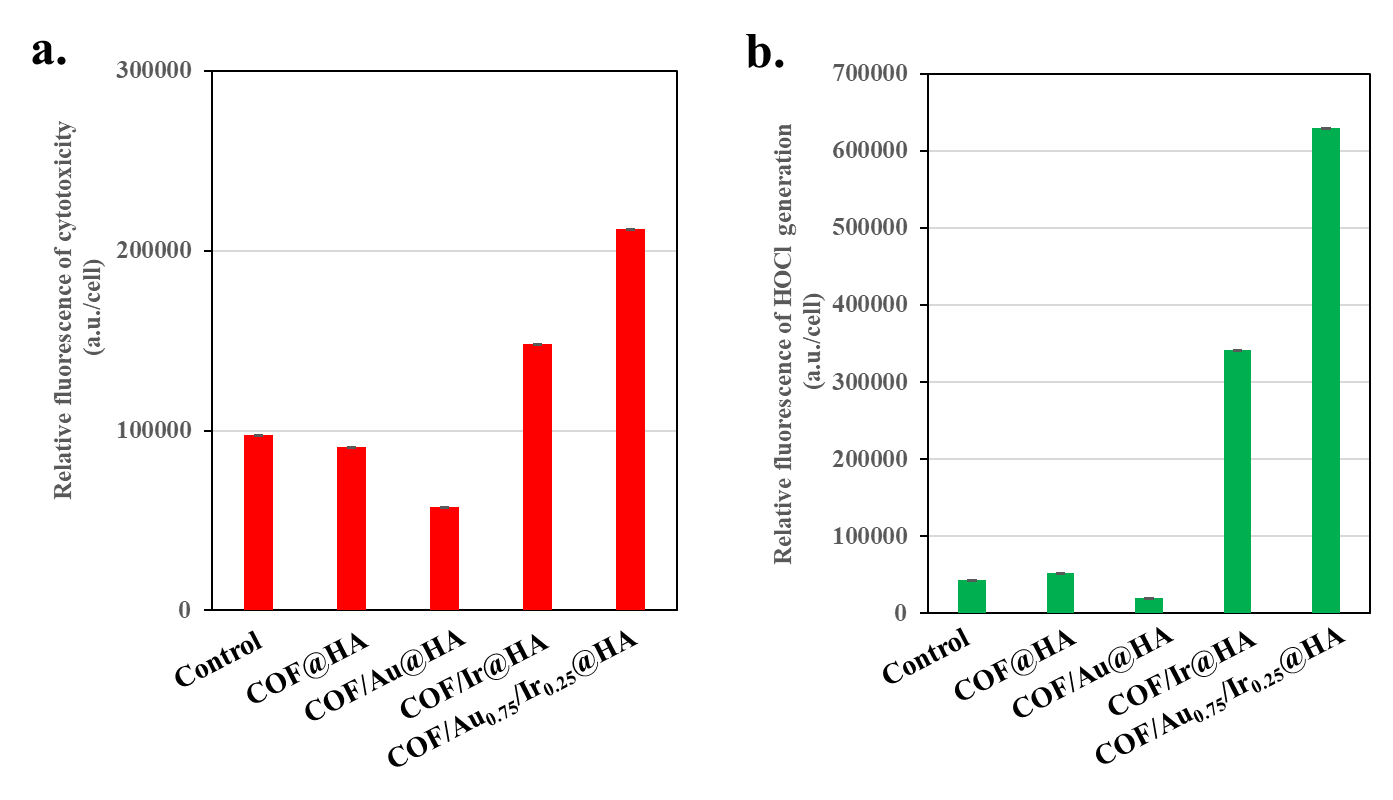


**Figure S29.** Quantitative fluorescence images of live and dead staining and RHS production. a) The red fluorescence of EthD-III for dead cells (corresponding to cytotoxicity). b) The green fluorescence of APF (corresponding to HOCl generation).


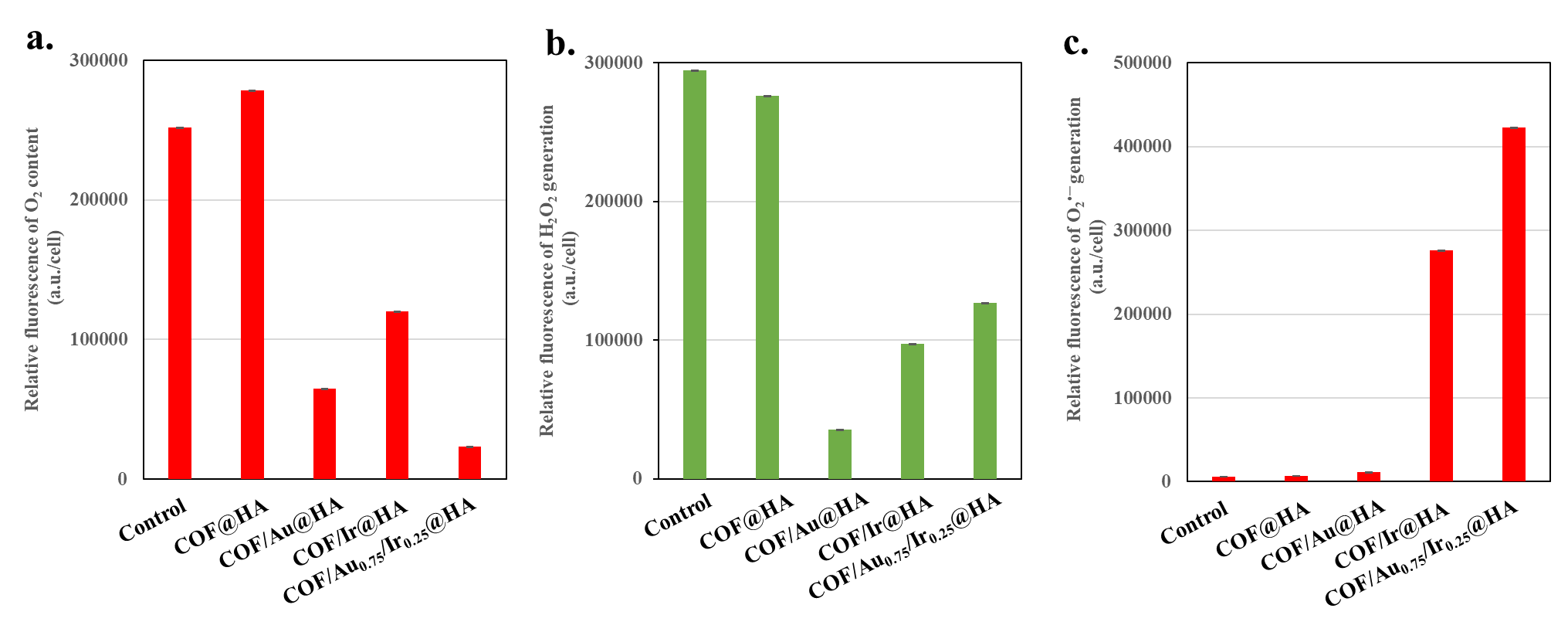


**Figure S30.** Quantification of fluorescence images. a) The decreased red fluorescence of [Ru(dpp)_3_]Cl_2_ (corresponding to O_2_ presence). b) The green fluorescence of H_2_O_2_ kit (corresponding to H_2_O_2_ content). c) The red fluorescence of DHE (corresponding to O_2_^•─^ presence).


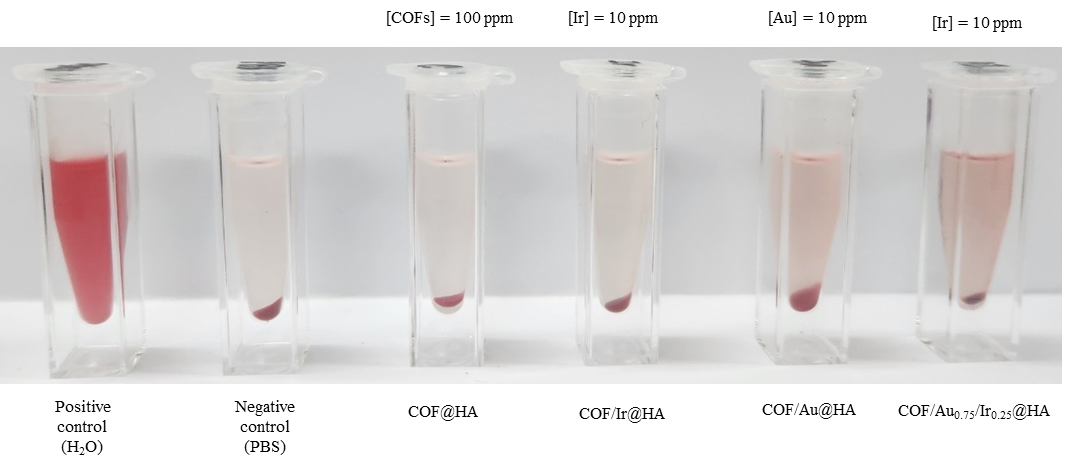


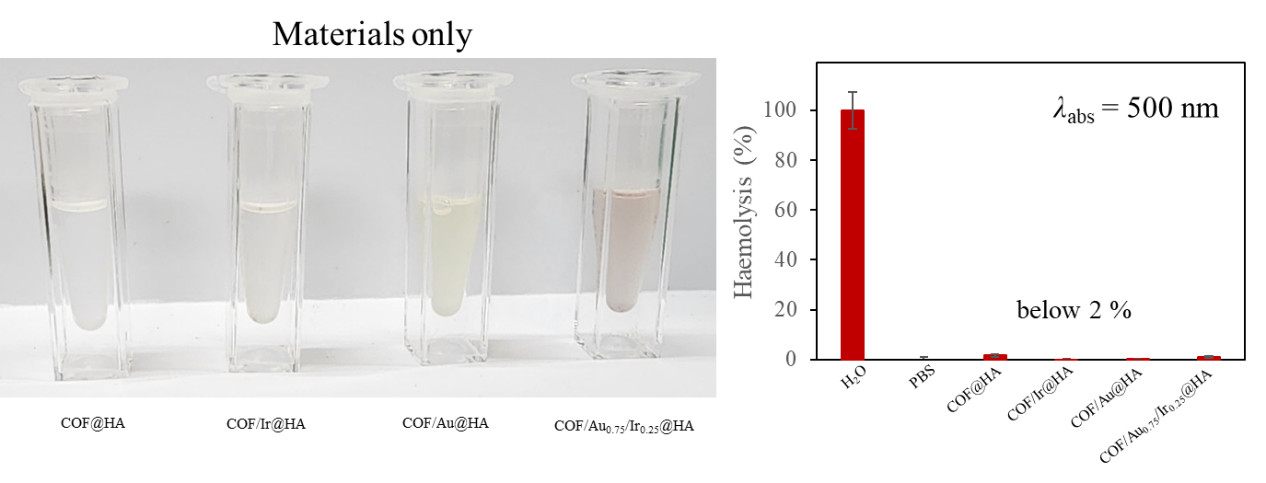


**Figure S31.** Analysis of hemolysis in blood containing 2% red blood cells from HA coating NPs at 0.5 h incubation. The slight coloration observed in the group with added materials originates from the NPs themselves, rather than from hemolysis. The effect of this coloration was deducted when calculating the hemolysis rate, resulting in a hemolysis rate of less than 2%.


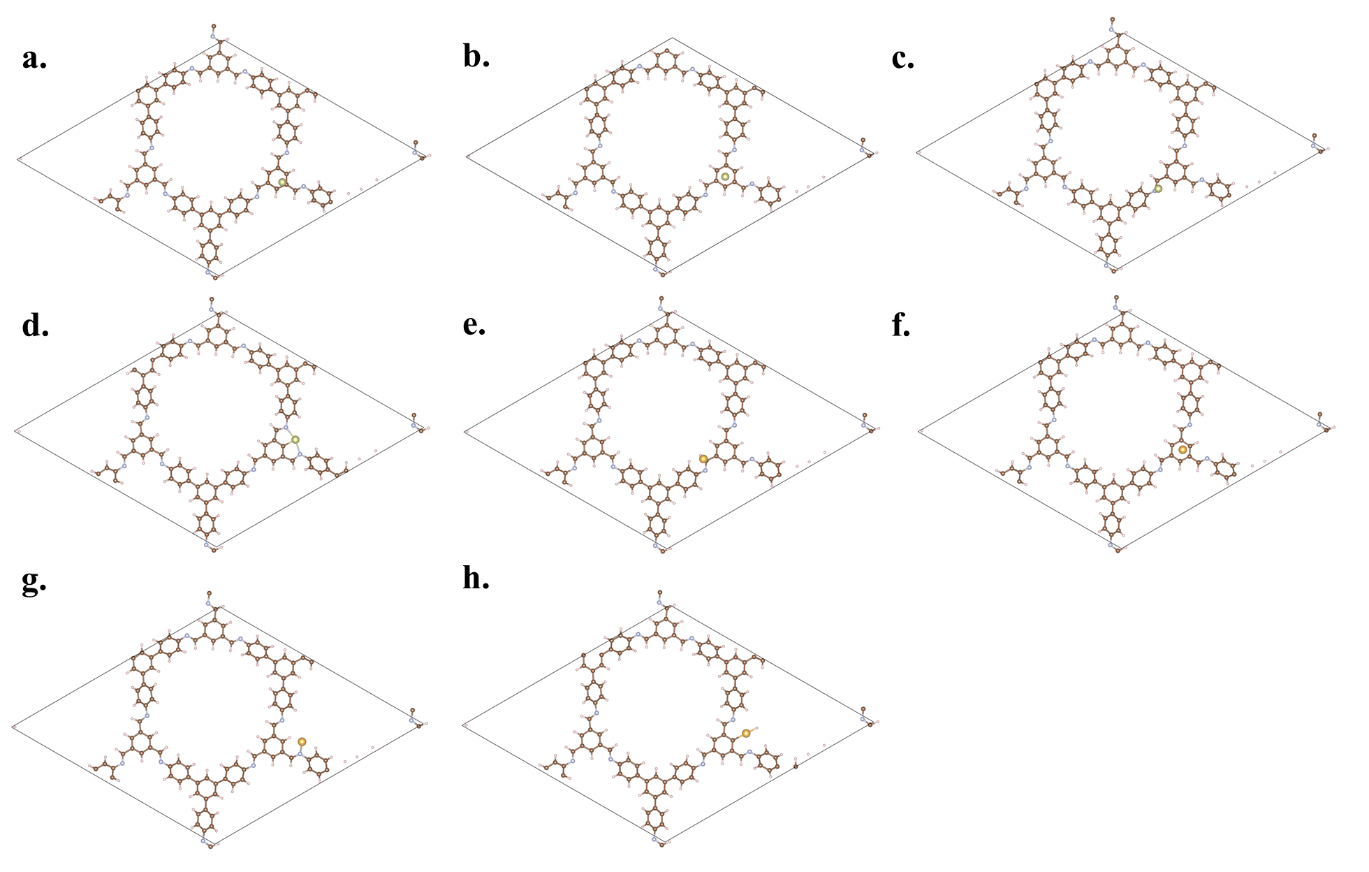


**Figure S32.** DFT optimized structures of Ir atom on the a) η^1^-C_6_H_5_, b) η^6^-C_6_H_5_, c) monodentate nitrogen, and d) bidentate nitrogen chelate sites; and Au atom on the e) η^1^-CN, f) η^6^-C_6_H_5_, g) monodentate nitrogen, and h) bidentate nitrogen chelate sites.

**Figure S33.** The analyzed body weight of C57BL/6 mice treated with COF/Au_0.75_/Ir_0.25_@HA during 7 days post i.v. injection (n=3). The error bars in represented mean ± SEM.


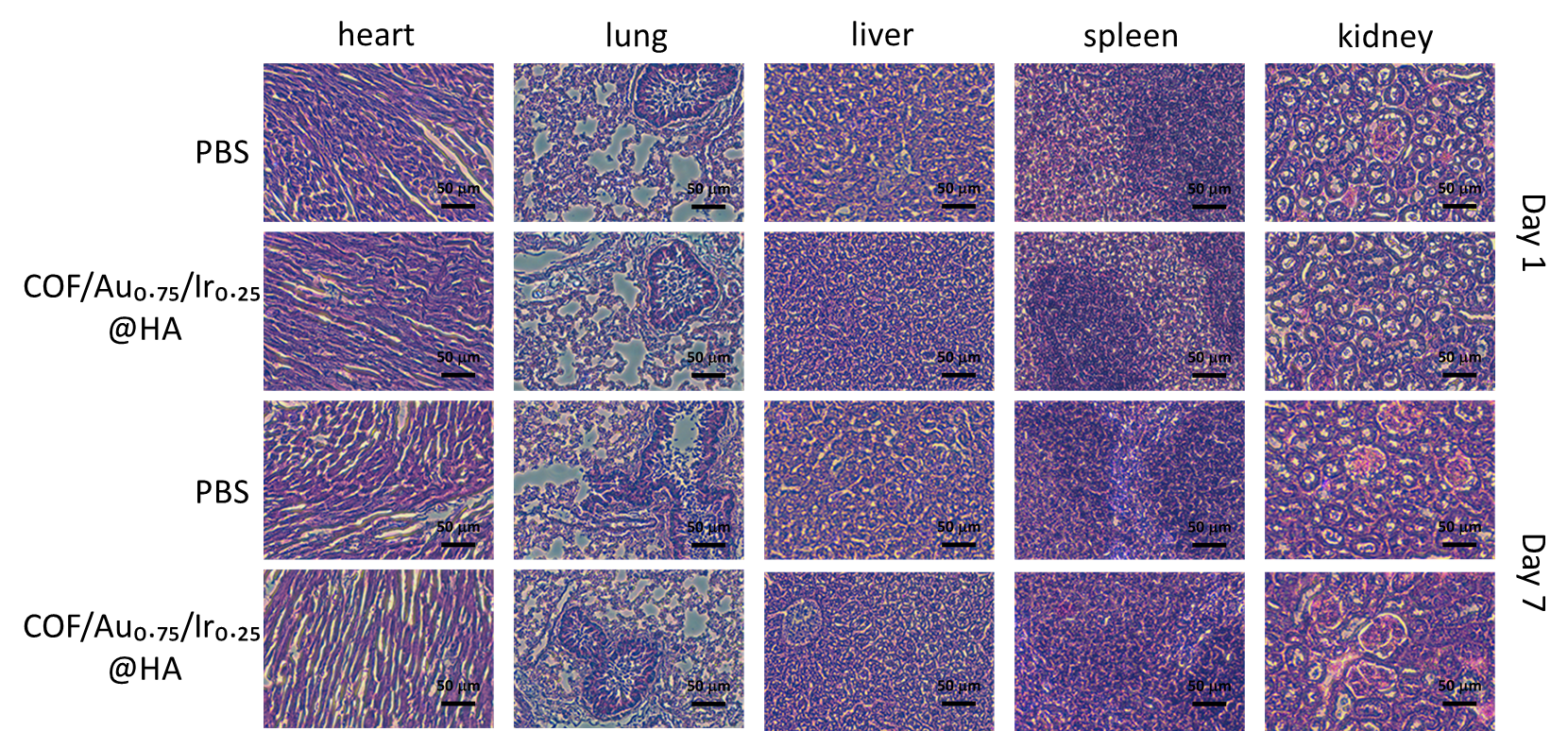


**Figure S34.** The histology morphology of each organ with PBS and COF/Au_0.75_/Ir_0.25_@HA NPs post-injection day 1 and day 7 were observed by H&E staining (scale bar, 50 μm).


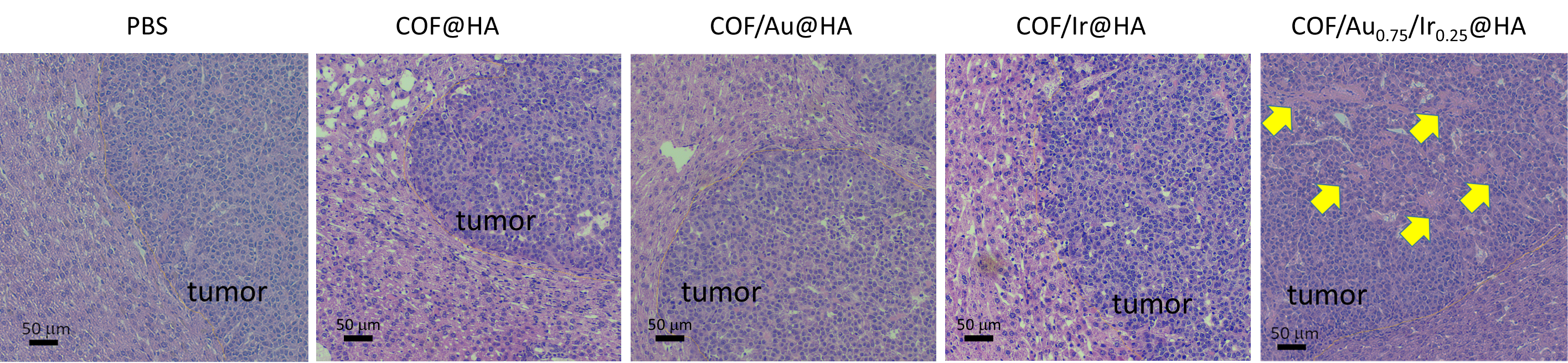


**Figure S35.** The histological analysis of tumor area of hepatocellular carcinoma reveals that COF/Au_0.75_/Ir_0.25_@HA treatment induced more extensive tumor necrosis compared to other groups. Scale bar = 50 μm
